# AI-driven discovery of probiotic gene modules and their functional impact in food and health

**DOI:** 10.3389/fmicb.2026.1870115

**Published:** 2026-07-22

**Authors:** Sumaya Alshatari, Małgorzata Ziarno

**Affiliations:** 1 Independent Researcher, Warsaw, Poland; 2 Department of Food Technology and Assessment, Institute of Food Science, Warsaw University of Life Sciences—SGGW (WULS—SGGW), Warsaw, Poland

**Keywords:** food matrix simulation, functional food design, graph neural networks, probiotic gene modules, protein language models, SCFA biosynthesis

## Abstract

**Introduction:**

How a probiotic works depends on its genes, its proteins, and the environment it lives in. Current knowledge gaps make it hard to predict how probiotics act in different foods or to find new, useful traits. To address this, we developed a new AI tool that analyzes probiotics from several angles—genes, proteins, and metabolism—to predict how they actually behave in different types of food.

**Methods:**

Using 156 fully annotated genomes, we identified 247 gene modules and uncovered latent functional clusters. Protein language models (ESM-2 and ProtT5) enabled the structural and biochemical characterization of 3,110 proteins, including lineage-restricted and previously unannotated sequences. Additionally, we simulated bacterial metabolic activity across different food matrices.

**Results:**

The framework successfully identified specific traits associated with stress tolerance, adhesion, short-chain fatty acid (SCFA) biosynthesis, and immunomodulation. Metabolic simulations demonstrated precisely where strains thrive and where metabolic bottlenecks occur, while models also revealed how these genetic traits might help boost the immune system or protect the gut lining. Notably, the AI’s predictions aligned closely with biological reality, confirming that the model captures meaningful insights rather than just statistical patterns.

**Discussion:**

This multi-scale framework offers a mechanistic basis for interpreting probiotic functional potential. By bridging genomic, proteomic, and environmental data, this approach supports the rational development and optimization of probiotic strains for specific food matrices.

## 1 Introduction

Probiotics are live microorganisms that, when administered in adequate amounts, confer health benefits on the host (Hill et al., 2014). Their use in functional foods and microbiome based interventions continues to expand, supported by evidence for roles in gastrointestinal homeostasis, immune modulation, and metabolic regulation (Sanders et al., 2019). Yet probiotic performance is highly context dependent. Survival during food processing and gastrointestinal transit, ecological persistence in the host, and downstream functional outcomes vary substantially across strains, hosts, and delivery matrices (Radicioni et al., 2019; Suez et al., 2019; Zmora et al., 2018). This variability limits the predictive value of strain selection strategies that rely primarily on taxonomy, empirical screening, or single trait assays.

From a systems perspective, probiotic function is not an intrinsic property of a genome alone, but an emergent phenotype shaped by interactions among genomic architecture, protein level functional capacity, and environmental constraints. Comparative studies have shown that host associated bacteria encode lineage specific adaptations affecting adhesion, stress tolerance, carbohydrate utilization, and metabolite exchange (Milani et al., 2017; Turroni et al., 2018). Short chain fatty acid production, one of the most biologically relevant outputs of probiotic metabolism, depends on the organization of both conserved and accessory pathways and is further modulated by nutrient availability and ecological context (Louis and Flint, 2017). Likewise, food matrices are not passive carriers. Dairy, plant based, and fermented environments impose distinct physicochemical and nutritional constraints that can reshape survivability, metabolic routing, and functional expression (Ranadheera et al., 2017). A predictive framework for probiotic biology therefore requires integration across genomic, proteomic, and metabolic scales.

Existing digital tools still struggle to bridge the gap between different biological scales, leaving much of the probiotic interaction puzzle unsolved. Genome annotation pipelines remain limited by incomplete reference databases and annotation transfer errors (Devos and Valencia, 2001). Homology based inference is especially weak for lineage restricted, poorly conserved, or previously uncharacterized proteins, which remain abundant in probiotic and microbiome associated genomes (Pasolli et al., 2019). At the same time, many machine learning approaches in microbiome research operate on single scale feature sets, such as taxonomic abundance, gene presence, or pathway summaries, without explicitly modeling how genomic modules interact with protein level traits and matrix dependent metabolism (Tierney et al., 2019; Topçuoğlu et al., 2020; Zmora et al., 2019). As a result, most available frameworks classify or rank strains retrospectively, but do not explain how strain specific functional outputs emerge under defined environmental conditions.

Recent advances in artificial intelligence and systems biology provide an opportunity to address these limitations. Deep learning methods can integrate heterogeneous biological data at a scale that was previously difficult to analyze coherently (Eraslan et al., 2019; Zitnik et al., 2019). Graph neural networks can capture higher order relationships among co-occurring functional modules (Reiser et al., 2022), while variational autoencoders can reveal latent organizational structure not evident from direct annotation alone (Kingma and Welling, 2014). Protein language models, including ESM2 and ProtT5, extend functional inference beyond classical homology by learning sequence encoded biochemical and structural regularities, enabling prediction for proteins that lack close annotated counterparts (Elnaggar et al., 2022; Lin et al., 2023). In parallel, genome scale metabolic modeling enables explicit representation of nutrient constrained phenotype, making it possible to test how food environments reshape flux distributions and functional potential.

These advances highlight the need for a new approach, one that explains exactly how probiotics function, rather than just predicting if they will work. Rather than asking only whether a strain contains known marker genes, a systems level approach can ask how genomic modules are organized, which latent protein functions they encode, how these features constrain metabolism across matrices, and whether the resulting multiscale signatures are associated with host relevant outputs. Such a framework is particularly useful for probiotic research, where function often depends on coordinated trait bundles rather than isolated determinants, and where experimental screening across strains, matrices, and phenotypes is labor intensive and difficult to scale.

Here, we present an AI driven, multiscale framework that integrates genomic modularity, transformer based protein inference, and matrix aware metabolic modeling to characterize probiotic function across food environments. Using 156 curated probiotic genomes, we identify 247 gene modules, infer protein level traits for both annotated and previously uncharacterized proteins, and simulate strain specific metabolic behavior under dairy, plant based, and fermented nutrient constraints. We then integrate genomic, proteomic, and metabolic features into supervised models linked to host relevant outcomes, including SCFA associated functional potential and IL10 related immunomodulatory signatures

In this study, we set out to answer four main questions. First, can probiotic genomes be resolved into recurrent functional modules and latent ecological architectures that are more informative than taxonomy alone? Second, do protein language models recover biologically meaningful functional signals from poorly annotated or lineage restricted proteins that would be missed by homology based pipelines? Third, does matrix constrained metabolic modeling reveal systematic rewiring of probiotic function across dairy, plant based, and fermented environments? Fourth, does integration of genomic, proteomic, and metabolic features improve prediction of host relevant phenotypes compared with any single layer alone?

We further tested three working hypotheses. We hypothesized that probiotic functional behavior is organized around multigene modules and latent systems level trait architectures rather than isolated marker genes. We hypothesized that protein language model derived features expand functional resolution by identifying cryptic proteins linked to stress tolerance, adhesion, and SCFA associated metabolism. Finally, we hypothesized that integrating genomic modularity, protein level inference, and matrix aware metabolic simulation would support more accurate and mechanistically interpretable prediction of host relevant probiotic phenotypes than single scale approaches. Together, this framework aims to move probiotic research from descriptive annotation toward predictive, systems level inference.

## 2 Methodology

**FIGURE 1:**
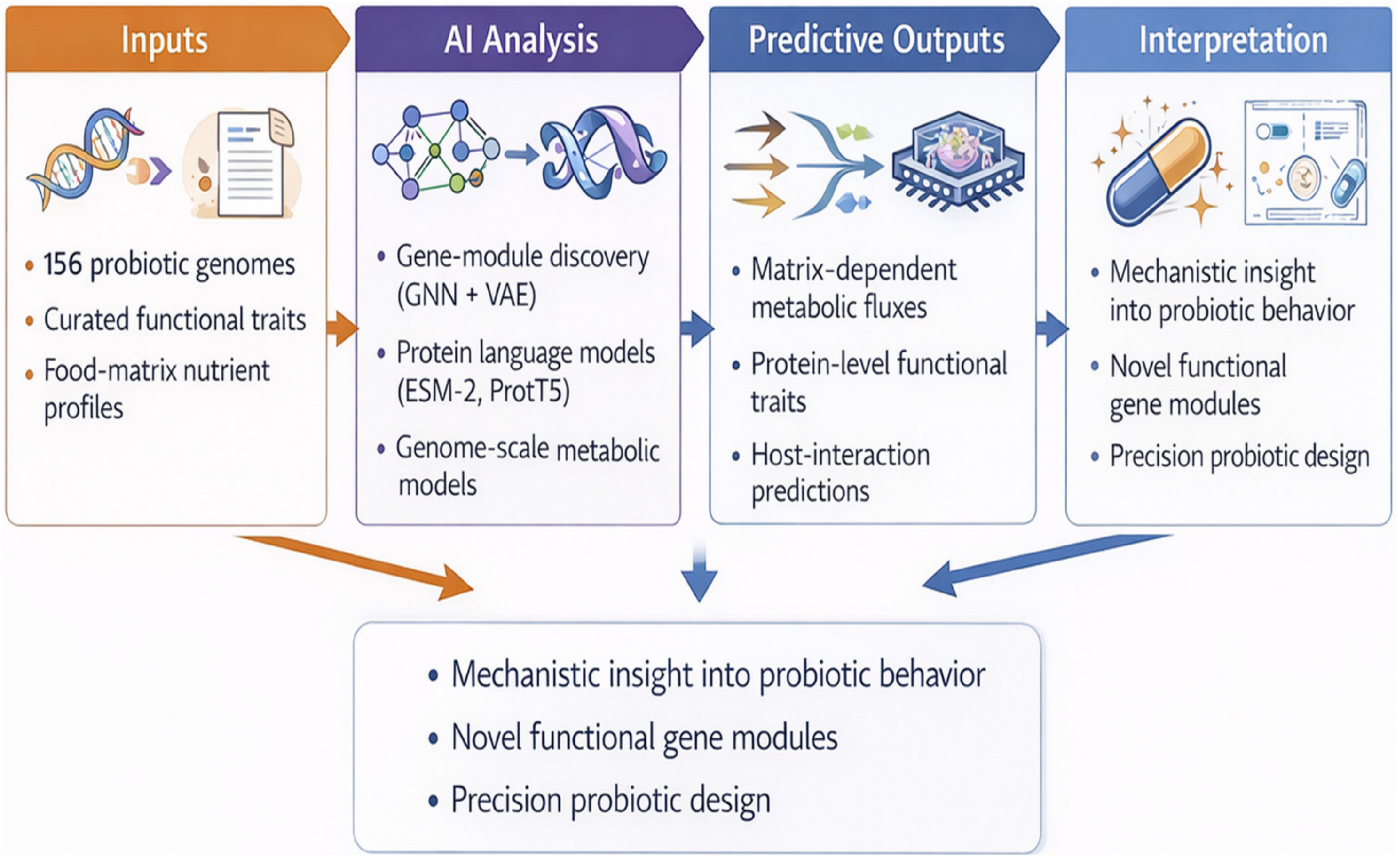
AI-driven multi-scale framework for predicting probiotic function across food matrices. The graphical abstract summarizes the integrated workflow combining curated genomic and trait inputs, AI-based gene-module discovery, transformer-based protein inference, and genome-scale metabolic modeling. These layers generate matrix-dependent metabolic fluxes, protein-level functional traits, and host-interaction predictions, enabling mechanistic insight into probiotic behavior and supporting precision probiotic design.

This study employs a multiphase, systems-level computational framework integrating literature-guided genome curation, comprehensive annotation, AI-driven gene-module discovery, transformer-based protein inference, genome-scale metabolic modeling, and supervised host-interaction prediction. Figure 1 presents a unified schematic of the computational pipeline, illustrating sequential progression from curated biological inputs through genomic, proteomic, and metabolic analyses to host-interaction prediction.

**FIGURE 2:**
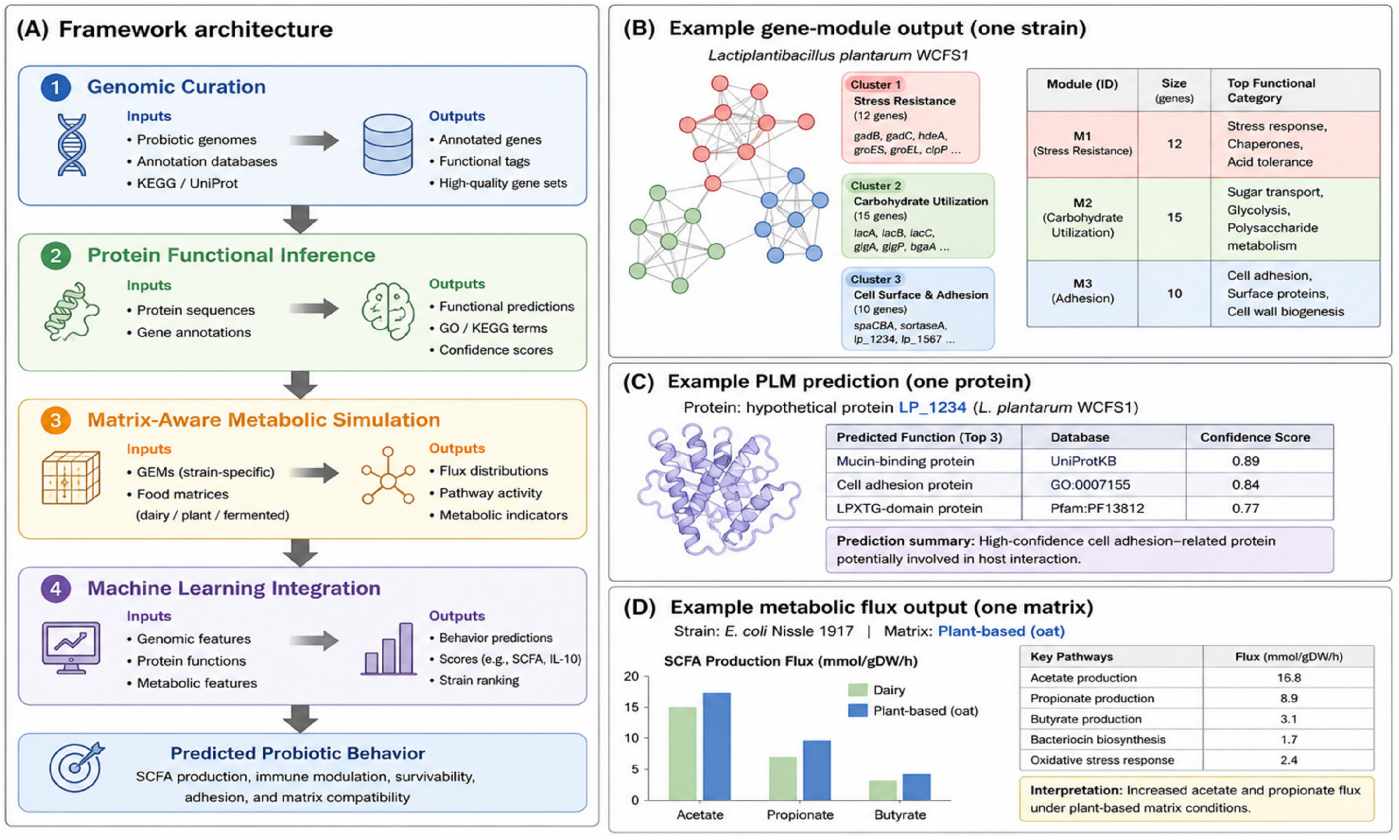
Overview of the AI-driven multi-scale framework and representative outputs. **(A)** Schematic representation of the four-stage computational workflow integrating genomic curation, protein functional inference, matrix-aware metabolic simulation, and machine-learning-based prediction to characterize probiotic behavior. **(B)** Representative gene-module output illustrating functional clustering and module organization within a probiotic strain. **(C)** Example protein language model (PLM)-based functional prediction showing inferred protein annotations and confidence scoring for an individual protein sequence. **(D)** Representative metabolic simulation output demonstrating matrix-specific flux predictions and pathway activity under defined environmental conditions. Together, these examples illustrate how multi-scale biological information is transformed into interpretable predictions for probiotic characterization and selection.

A schematic overview of the full AI-driven framework, including data inputs, analytical layers, and representative outputs, is provided in Figure 2. Figure 2A summarizes the overall architecture of the AI-driven framework, showing how curated genomic inputs progress through annotation, protein-level inference, metabolic simulation, and machine-learning integration. Figure 2B illustrates a representative gene-module output, highlighting functionally coherent clusters such as stress-response, carbohydrate-utilization, and adhesion-related modules identified in *Lactiplantibacillus plantarum* WCFS1. Figure 2C presents an example of protein-level functional inference using transformer-based language models, demonstrating how previously unannotated proteins (e.g., LP_1234) receive high-confidence predictions related to adhesion and host interaction. Figure 2D shows an example metabolic simulation output, comparing short-chain fatty acid fluxes across food matrices and illustrating matrix-dependent metabolic shifts in *E. coli* Nissle 1917.

### 2.1 Systematic literature screening and genome curation

**FIGURE 3:**
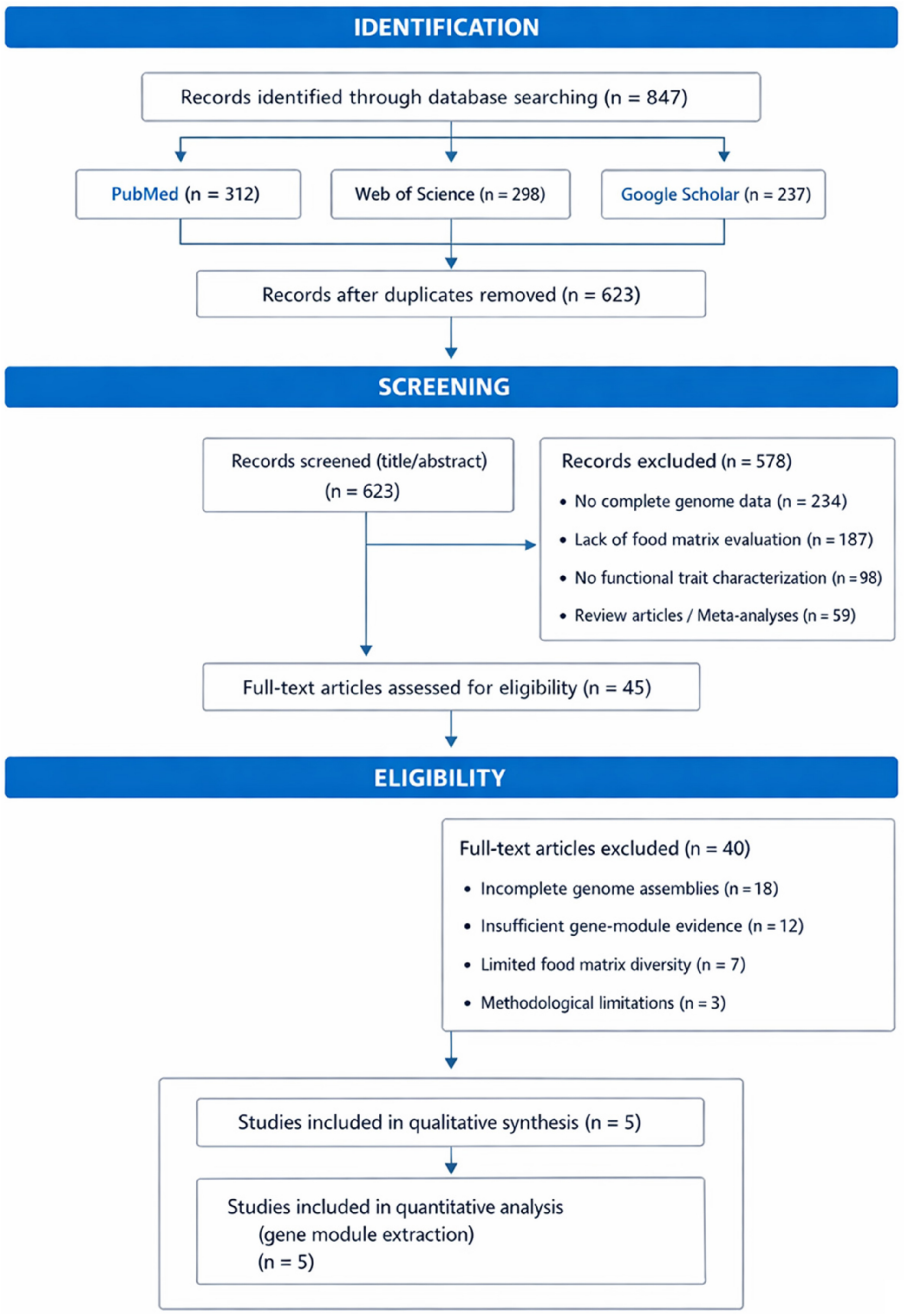
PRISMA flowchart for systematic literature screening and study selection process. The diagram illustrates the systematic approach used to identify and select peer-reviewed studies for literature-guided genome curation.

To ensure our foundation was built on high-quality evidence, we applied the PRISMA framework to systematically filter for the most rigorous and relevant studies (Moher et al., 2009). The complete screening workflow is illustrated in Figure 3, which presents the PRISMA flowchart documenting the systematic approach used for study identification, screening, eligibility assessment, and final inclusion. These studies collectively represented advanced research on *Lactiplantibacillus plantarum*, *Bifidobacterium longum*, *Escherichia coli* Nissle 1917, and engineered probiotic systems (Supplementary Table S1).

#### 2.1.1 Search strategy and database selection

Comprehensive searches were conducted across three databases (PubMed, Web of Science, Google Scholar) using search terms: (“probiotic” OR “Lactiplantibacillus” OR “Bifidobacterium” OR “*Escherichia coli* Nissle”) AND (“genome” OR “genomic”) AND (“food matrix” OR “dairy” OR “plant-based” OR “fermentation”) AND (“functional” OR “phenotype” OR “trait”) for publications from January 2017 to December 2023.

#### 2.1.2 Inclusion and exclusion criteria

Studies were required to meet four inclusion criteria: (1) complete genome sequences with > 95% assembly completeness and coordinated annotations, (2) experimentally characterized probiotic traits with quantitative measurements, (3) documented gene-module or operon-level functional evidence supported by transcriptomic, proteomic, or phenotypic data, (4) explicit evaluation of food-matrix effects across at least two different substrate types (dairy, cereal, soy, plant-based materials).

#### 2.1.3 Screening protocol

Initial screening involved title and abstract review by two independent reviewers, followed by full-text assessment for eligibility. Disagreements were resolved through consensus discussion. Studies were excluded for incomplete genome assemblies ( < 95% completeness), insufficient gene-module evidence, limited food-matrix diversity ( < 2 matrix types), or methodological limitations. A total of 847 records were retrieved, 623 screened after duplicate removal, 45 assessed for eligibility, and 5 studies met all inclusion criteria.

#### 2.1.4 Data extraction framework

Selected studies were processed using a structured extraction schema capturing strain identity and genomic architecture, validated gene-module composition and operon organization, matrix-specific functional modulation across substrates, and host-relevant physiological outcomes including cytokine responses and epithelial barrier effects. This systematic approach ensured that only methodologically rigorous studies contributed to the foundation of computational analysis.

#### 2.1.5 The computational workflow consists of five stages

(1) genome annotation and module detection, (2) protein-level functional inference using PLMs, (3) matrix-aware metabolic simulation, (4) multi-scale feature integration, and (5) supervised learning with SHAP-based interpretability. A complete step-by-step pipeline, including Snakemake, Nextflow, and CWL workflows, is provided in the Zenodo repository.^1^

To enhance methodological transparency and ensure full reproducibility, we expanded all analytical descriptions to include explicit software versions, parameter settings, and quality thresholds. Genome inclusion required > 95% completeness (CheckM v1.2.0), high assembly quality (QUAST v5.2.0), and species-level confirmation using FastANI v1.33 and GTDB-Tk v2.1.1. Module detection thresholds were defined using operon proximity, KEGG/RAST functional coherence, evolutionary co-occurrence (DOOR v3), and regulatory linkage evidence. All annotation, clustering, protein-inference, and metabolic-modeling tools are reported with version numbers, and their key parameters are described in sections 2.2–2.6 to allow replication without consulting the repository. These additions address the need for methodological clarity and ensure that each step of the pipeline can be reproduced directly from the manuscript.

### 2.2 Whole-genome annotation and functional module categorization

This section describes the genome annotation and module-definition procedures used to construct the genomic feature layer. Genome Selection and Quality Assessment: A total of 156 probiotic genomes representing *Lactiplantibacillus*, *Bifidobacterium*, and *Escherichia coli* Nissle lineages were selected based on completeness, assembly quality, and availability of supporting phenotypic information. Genomes were obtained from NCBI RefSeq, GenBank, or curated literature sources. Quality assessment was performed using CheckM v1.2.0 (Parks et al., 2015) for completeness estimation, QUAST v5.2.0 (Gurevich et al., 2013) for assembly statistics, FastANI v1.33 (Jain et al., 2018) for species-level identification, and GTDB-Tk v2.1.1 (Chaumeil et al., 2022) for taxonomic classification.

Multi-Platform Annotation Pipeline: To maximize annotation depth and reduce tool-specific bias, three complementary annotation systems were integrated using a consensus-based strategy. Prokka v1.14.6 (Seemann, 2014) provided rapid prokaryotic genome annotation, RASTtk v2.0 (Brettin et al., 2015) delivered subsystem-based functional classification, and KOfamScan v1.3.0 (Aramaki et al., 2020) assigned KEGG orthology identifiers. Additional functional annotations were obtained from UniProt/Swiss-Prot 2023_04 (The UniProt Consortium, 2023), Pfam v35.0 (Mistry et al., 2021), BLAST+ v2.13.0 (Camacho et al., 2009), and InterProScan v5.63-95.0 (Jones et al., 2014).

Gene Module Definition and Validation Functional modules were defined using standardized criteria incorporating: (i) genomic proximity within operons or conserved clusters identified by Operon-mapper and Roary v3.13.0 (Page et al., 2015), (ii) functional coherence based on KEGG pathways and RAST subsystems, (iii) evolutionary co-occurrence across phylogenetically diverse strains using DOOR v3 (Page et al., 2015), and (iv) regulatory linkage evidence from transcriptomic or operon-level studies. Modules were assigned to five functional domains: adhesion and colonization, stress resistance, short-chain fatty acid biosynthesis, antimicrobial activity, and immunomodulation. Pathway mapping used MetaCyc v26.0 (Caspi et al., 2020) for metabolic pathway annotation.

The complete list of 247 gene modules, including functional annotations and representative genes, is provided in Supplementary Table S2.

### 2.3 AI-powered clustering and trait inference

This subsection outlines the AI-based methods used to identify latent functional structure among gene modules. Graph Neural Network Implementation Genome-wide co-occurrence patterns were modeled using graph neural networks implemented in PyTorch v2.1.0 (Paszke et al., 2019) and PyTorch-Geometric v2.4.0 (Fey and Lenssen, 2019). Each gene module was represented as a node with edges encoding co-presence frequencies, subsystem associations, and KEGG-derived functional linkages. Graph construction incorporated phylogenetic distance, genomic synteny, and functional similarity metrics.

Variational Autoencoder Analysis Variational autoencoders were trained using PyTorch backend to embed module features into low-dimensional latent space capturing nonlinear structure. Architecture comprised encoder and decoder networks with regularization through KL divergence terms. Latent dimensions were optimized through cross-validation to balance reconstruction accuracy and dimensional compression.

Transformer-Based Attention Mechanisms Attention models were implemented using HuggingFace Transformers v4.36.0 (Wolf et al., 2020) to identify modules with strong cross-strain functional coherence. Multi-head attention mechanisms captured long-range dependencies between functionally related modules across genomic contexts.

Dimensionality Reduction and Clustering Integrated GNN-VAE-transformer outputs were visualized using UMAP-learn v0.5.4 (McInnes et al., 2018) for nonlinear dimensionality reduction. Clustering employed scikit-learn v1.3.1 (Pedregosa et al., 2011) algorithms including k-means, hierarchical clustering, and Consensus ClusterPlus v1.58.0 (Wilkerson and Hayes, 2010) for robust cluster identification and validation.

### 2.4 Protein-level structural function mapping via language models

This section details the protein-level functional inference pipeline based on transformer-based language models. Amino acid sequences were extracted from annotated genomes using BioPython v1.81 (Cock et al., 2009). Preprocessing included signal peptide prediction with SignalP v6.0 (Teufel et al., 2022), transmembrane domain identification using TMHMM v2.0 (Krogh et al., 2001), low-complexity region masking with the SEG algorithm (Wootton and Federhen, 1996), and redundancy reduction using CD-HIT v4.8.1 (Fu et al., 2012).

Two complementary protein language models were employed. ESM-2 650M (Lin et al., 2023) generated structure-aware embeddings encoding secondary and tertiary features relevant to catalytic activity, fold stability, and surface accessibility. ProtT5 XL UniRef50 (Elnaggar et al., 2022) produced high-dimensional contextual embeddings that capture physicochemical signatures, conserved motifs, and functional patterns. Embeddings from both models were concatenated into a single feature matrix for downstream analysis. All PLM-based protein annotations and prediction confidence scores are summarized in Supplementary Table S3.

Functional categories were derived from KEGG Release 107.0 (Kanehisa et al., 2017), RAST subsystems, and Prokka annotations, supplemented with literature-validated labels. Classification employed multiple algorithms, including random forests, gradient boosting, and neural networks, implemented in scikit-learn. Model validation used stratified cross-validation, with performance assessed using accuracy, precision, recall, and F1-score metrics. Structural validation is used by the FFAS server (Jaroszewski et al., 2009) for fold recognition and functional annotation transfer.

### 2.5 Matrix-specific simulation of metabolic behavior

This subsection describes the reconstruction and simulation of genome-scale metabolic models under matrix-specific constraints. Genome-Scale Model Reconstruction Genome-scale metabolic models were reconstructed using CarveMe v1.5.1 (Machado et al., 2018) with universal biochemical reaction database and strain-specific gene annotations. Models underwent standardized curation including gap-filling, thermodynamic consistency checking, dead-end metabolite removal, and verification of lactic-acid-bacteria-specific pathways. Model validation employed MEMOTE v0.13.0 (Lieven et al., 2020) for quality assessment and libSBML v5.20.2 (Bornstein et al., 2008) for SBML format compliance.

Environmental constraint definition matrix-specific nutrient constraints were derived from experimentally reported compositions. Dairy matrices were characterized by high lactose availability, elevated buffering capacity, and moderate amino acid content. Plant-based matrices incorporated complex polysaccharides, fermentable fibers, and lower buffering capacity. Fermented matrices included organic acids, redox-active metabolites, and reduced sugar availability. Constraints were implemented as upper bounds on substrate uptake reactions defining feasible metabolic space.

Flux balance analysis protocol metabolic simulations employed COBRApy v0.27.0 (Ebrahim et al., 2013) with GLPK v5.0 (Makhorin, 2012) linear programming solver. Flux balance analysis used biomass maximization objective to generate steady-state flux distributions for each strain-matrix combination. Alternative objectives included SCFA production maximization and ATP yield optimization.

Metabolic pathway analysis flux distributions were mapped to metabolic pathways and gene modules through gene-protein-reaction associations. Pathway enrichment analysis identified matrix-favored metabolic routes using OptGP algorithm (Megchelenbrink et al., 2014) for optimal pathway sampling and flux variability analysis for robustness assessment.

### 2.6 Data integration and supervised learning framework

This section explains how genomic, proteomic, and metabolic features were integrated into a unified dataset for supervised learning. Multi-Layer Feature Architecture Three feature categories were constructed: (1) Module-level genomic features comprising binary presence/absence matrices with conservation scores and co-occurrence frequencies, (2) Protein-level functional signatures from transformer embeddings processed through PCA dimensionality reduction, (3) Matrix-aware metabolic flux features incorporating normalized pathway values and activation scores.

Data integration and quality assurance feature integration employed standardized preprocessing with z-score normalization and k-nearest neighbors imputation. Quality control included correlation analysis, variance filtering, and outlier detection using isolation forest algorithm. Feature selection employed univariate statistical testing, recursive feature elimination, and L1 regularization.

Supervised learning implementation training datasets included experimentally validated probiotic-host interaction data documenting cytokine responses, epithelial barrier effects, and SCFA-mediated outcomes. Multiple algorithms were evaluated: XGBoost v1.7.3 (Chen and Guestrin, 2016), Random Forest from scikit-learn, and neural networks in TensorFlow v2.11.0 (Abadi et al., 2016). Training employed stratified k-fold cross-validation with hyperparameter optimization via Optuna v3.1.0 (Akiba et al., 2019).

Model validation and interpretation validation employed independent test sets, temporal validation, and biological validation through literature comparison. Feature importance analysis used SHAP v0.41.0 (Lundberg and Lee, 2017) for model interpretability and Captum v0.6.0 (Kokhlikyan et al., 2020) for attention weight visualization.

All computational workflows employed Git v2.34.1 (Chacon and Straub, 2014) for version control and Docker v20.10.17 (Merkel, 2014) for containerization to ensure reproducibility. Data processing utilized Python v3.10.12 (Van Rossum and Drake, 2009), NumPy v1.26.0 (Harris et al., 2020), Pandas v2.1.0 (McKinney, 2010), and Matplotlib v3.8.0 (Hunter, 2007) for visualization.

To strengthen statistical transparency, we expanded the description of all evaluation metrics used across clustering, dimensionality reduction, and predictive modeling. Clustering stability was assessed using silhouette scores, consensus clustering indices, and VAE reconstruction error. Predictive models were evaluated using stratified k-fold cross-validation, with performance quantified using AUC-ROC, precision, recall, F1-score, and precision-recall curves. Agreement between predicted and observed SCFA values was evaluated using Spearman correlation with two-tailed significance testing (α = 0.05). For IL-10 classification, statistical significance of model predictions was assessed using McNemar’s test. These additions provide clear quantitative evaluation metrics and clarify how statistical significance was determined throughout the analytical workflow.

### 2.7 Data partitioning and independence of validation datasets

To ensure rigorous model evaluation and minimize information leakage, the analytical workflow was divided into non overlapping discovery, training, held out test, and external validation components. The discovery set consisted of 156 curated probiotic genomes, which were used for genome annotation, gene module identification, protein feature extraction, and matrix specific metabolic simulations. These genomes provided the biological foundation for constructing the multiscale feature space but were not, by themselves, treated as supervised outcome labels.

For supervised host interaction modeling, a separate labeled dataset was assembled from experimentally characterized probiotic host interaction studies reporting cytokine responses, epithelial barrier effects, and related functional outcomes. After quality control and label harmonization, the final supervised dataset was divided into stratified training and held out test partitions in an 80:20 ratio, with exact record counts provided in Supplementary Table S6. All hyperparameter optimization, feature selection, and model fitting procedures were performed exclusively within the training partition, with stratified 5 fold cross validation applied only to the training data.

External validation was performed using independent literature derived datasets that were not used for supervised model training. For SCFA validation, the external benchmark comprised 45 matched strain matrix pairs from controlled fermentation studies spanning dairy and plant based environments. Predicted acetate, propionate, and butyrate fluxes were aligned to experimental measurements only after the computational models had been finalized. For host interaction validation, IL10 related assay records used for final biological benchmarking were also maintained separately from the training workflow wherever independent studies were available.

To preserve independence across analytical stages, overlap was explicitly excluded at three levels. First, strain overlap was not allowed between training and held out test partitions. Second, study overlap was controlled by assigning records derived from the same publication, experiment, or assay series to a single partition only, thereby preventing closely related observations from being split across training and evaluation datasets. Third, matrix overlap was controlled during external SCFA validation by ensuring that identical strain matrix observations used for literature benchmarking were not included in model training. When the same strain appeared in multiple food environments, each strain matrix pair was treated as a distinct context dependent observation, but partitioning was performed so that no identical strain study matrix combination appeared in more than one dataset. Together, these constraints ensured that reported performance reflected generalization across biologically meaningful units rather than partial duplication of closely related records.

### 2.8 Validation strategy

This subsection outlines the validation strategy used to assess the biological relevance of model predictions. To ensure methodological robustness, all predictive components of the framework were validated using independent experimental datasets and literature-derived measurements spanning two functional domains with well-established quantitative benchmarks: short-chain fatty acid (SCFA) production and IL-10–associated immunomodulation. For SCFA validation, a curated dataset was assembled from published fermentation studies reporting quantitative acetate, propionate, and butyrate concentrations for *Lactiplantibacillus*, *Bifidobacterium*, and *Escherichia coli* Nissle strains grown in defined dairy or plant-based matrices. Studies were included only when they provided controlled fermentation conditions, strain-level identification, quantitative metabolite measurements, and at least two biological replicates. This process yielded 45 matched strain-matrix pairs (Supplementary Table S5). Predicted SCFA fluxes from matrix-specific metabolic simulations were aligned with experimental measurements based on strain identity, matrix type, and comparable fermentation time points. Agreement between predicted and observed values was quantified using Spearman correlation, per-metabolite correlation, and precision-recall analysis to assess the model’s ability to identify SCFA-enriched conditions. Statistical significance was evaluated using two-tailed tests with α = 0.05.

Validation of host-interaction predictions used in vitro cytokine assay data from peer-reviewed studies reporting IL-10 induction by well-characterized probiotic strains. Strains were classified as high or low IL-10 inducers using thresholds defined in the original studies (Supplementary Table S6). Performance was assessed using AUC-ROC, sensitivity, specificity, precision, and recall, with McNemar’s test applied to evaluate classification significance. Model interpretability was examined using SHAP (SHapley Additive exPlanations), enabling identification of genomic, proteomic, and metabolic features contributing to IL-10 predictions and providing mechanistic insight into model behavior.

All validation datasets, scripts, and statistical workflows are available in the Zenodo archive (see text footnote 1) under the validation/ directory, ensuring full transparency.

### 2.9 Reproducibility

All analyses in this study were designed to be fully reproducible through transparent data organization, version-controlled code, and openly accessible computational workflows. The complete framework, including genome curation, module detection, protein language model inference, metabolic simulation, and supervised learning, is available in both a public GitHub repository and a permanent Zenodo archive.

*GitHub repository*: https://github.com/somiasammer-eng/AI-Driven-Multi-Scale-Framework-for-Probiotic-Gene-Module-Discovery.git.

*Zenodo archive (citable snapshot)*: https://doi.org/10.5281/zenodo.20530781.

The archive contains:

- curated genomic datasets (156 genomes)
- gene-module matrices (247 modules)
- protein embeddings (ESM-2 and ProtT5)
- strain-specific genome-scale metabolic models (SBML)
- matrix-specific flux simulations (10,248 profiles)
- integrated AI-ready feature matrices
- trained machine-learning models
- benchmarking datasets
- figure-generation scripts
- workflow documentation and environment files

Reproducibility is ensured through the inclusion of Conda environment files, Python dependency lists, Docker and Singularity containers, and workflow scripts (Snakemake, Nextflow, CWL). All analyses were executed using fixed software versions, and random seeds were set for all machine-learning models to ensure deterministic behavior. Detailed execution instructions are provided in the README.md and docs/directories.

### 2.10 Model configuration, data filtering, and evaluation procedures

Predictive analyses were conducted using standardized preprocessing, explicit feature-selection procedures, and controlled validation workflows to ensure methodological transparency and reproducibility. Genomic, proteomic, and metabolic features were first filtered for prevalence, reduced using principal component analysis, and normalized prior to model training. Missing values were imputed using k-nearest neighbors, and outliers were identified using an isolation-forest approach.

Feature selection followed a multi-stage strategy combining univariate statistical testing, recursive feature elimination, and L1-regularized logistic regression. Only features consistently selected across methods were retained to minimize model-specific bias.

Supervised learning models, including XGBoost, random forests, and neural networks, were trained with stratified 5-fold cross-validation applied to all classification tasks. All hyperparameter tuning was performed exclusively on the training set to prevent information leakage. Model performance was evaluated using AUC-ROC, sensitivity, specificity, precision, recall, and F1-score for classification tasks, and correlation-based metrics and error estimates for regression tasks.

Model interpretability was assessed using SHAP to quantify the contribution of genomic, proteomic, and metabolic features to individual predictions. Confusion matrices and ROC curves were generated for the held-out test set to visualize classification performance and threshold-dependent behavior.

All hyperparameters, filtering thresholds, and full evaluation outputs are provided in Supplementary Table S4 and the Zenodo archive (see text footnote 1), enabling complete replication of the predictive analyses.

### 2.11 Detailed parameters and settings

#### 2.11.1 Software and computing environment

All analyses were performed using Python 3.10 on Ubuntu 20.04. Key software versions included Prokka v1.14.6, RASTtk v2.0, KOfamScan v1.3.0, PyTorch v2.1, scikit-learn v1.3, COBRApy v0.27, and UMAP-learn v0.5.4.

#### 2.11.2 Genome inclusion criteria

Genomes were retained if completeness was ≥ 95% and contamination ≤ 5% (CheckM v1.2.0), with N50 ≥ 50 kb and assembly size within lineage-typical ranges. Assemblies containing more than 500 contigs or showing ambiguous taxonomic classification were excluded.

#### 2.11.3 Module detection parameters

Gene modules were identified using hierarchical clustering with Ward linkage and a Jaccard distance threshold of 0.3. Modules were required to contain at least three genes, exhibit genomic proximity of ≤ 10 kb, and show functional coherence supported by KEGG or RAST annotations.

#### 2.11.4 Protein language model settings

ESM-2 (650M) and ProtT5-XL-UniRef50 were executed using default inference parameters. The resulting embeddings (1,280- and 1,024-dimensional, respectively) were concatenated to generate unified feature matrices for downstream classification.

#### 2.11.5 Machine learning parameters

Supervised models were trained using an 80/20 train–test split and evaluated with 5-fold cross-validation. Performance metrics included accuracy, precision, recall, F1-score, and AUC-ROC.

#### 2.11.6 Metabolic model reconstruction

Genome-scale metabolic models were reconstructed using CarveMe v1.5.1 and evaluated with MEMOTE. Matrix-specific nutrient constraints were applied based on literature-derived uptake rates.

#### 2.11.7 Statistical analysis

Comparisons were performed using Student’s *t*-tests or Mann–Whitney U tests depending on normality (Shapiro–Wilk). Multiple testing was corrected using the Benjamini–Hochberg procedure. Predictive models were evaluated using accuracy, precision, recall, F1-score, and AUC-ROC under 5-fold cross-validation.

## 3 Results

### 3.1 Expanded genomic dataset reveals robust modular architecture

**FIGURE 4:**
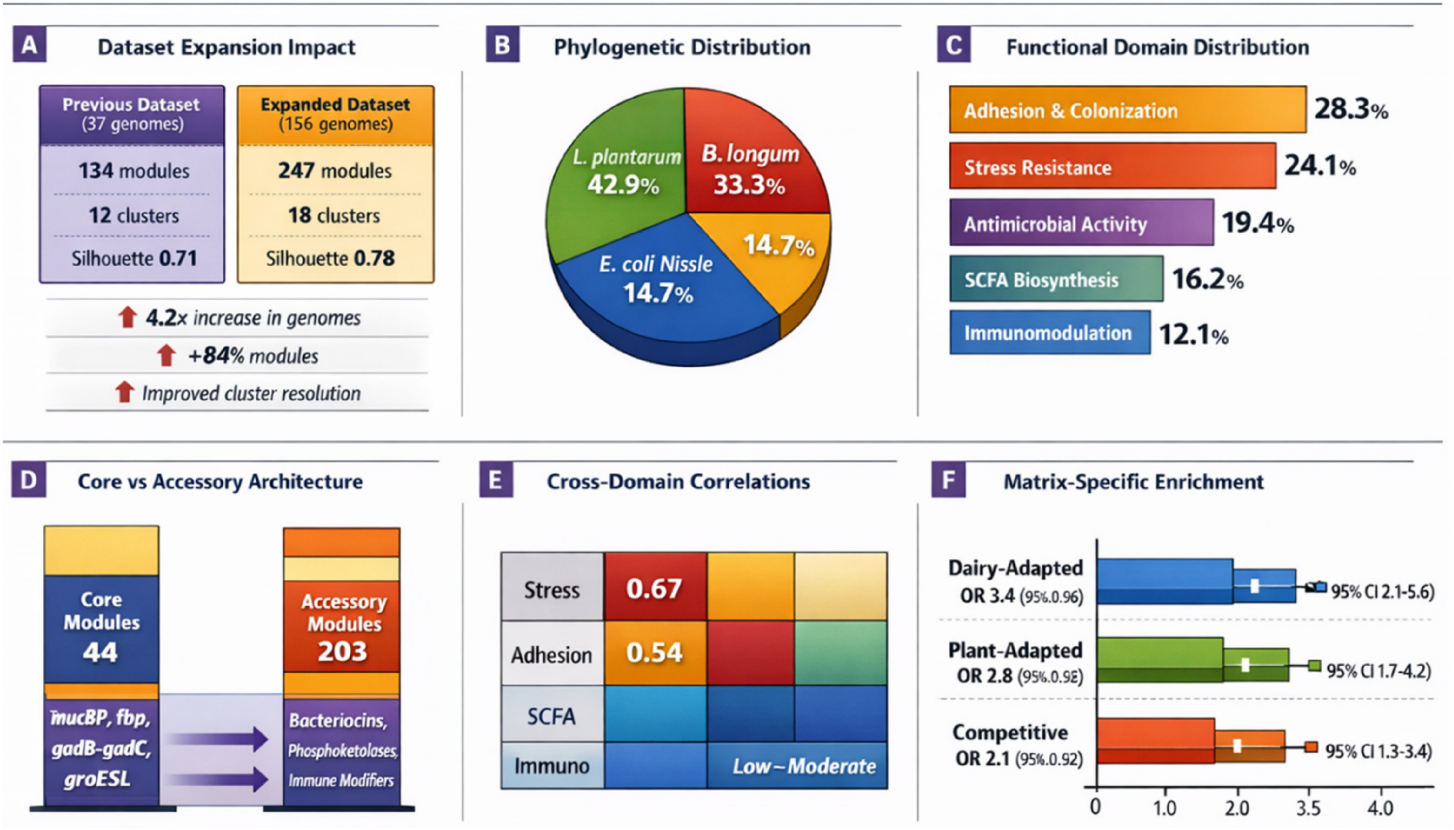
An expanded genomic dataset reveals a robust modular architecture across 156 probiotic genomes. **(A)** Expansion from 37 to 156 genomes increased the number of detected gene modules (134–247; +84%) and functional clusters (12–18), improving clustering robustness (silhouette coefficient from 0.71 to 0.78). **(B)** Phylogenetic composition of the dataset, dominated by *Lactiplantibacillus plantarum* (42.9%) and *Bifidobacterium longum* (33.3%), followed by *Escherichia coli* Nissle 1917 (14.7%) and other species (9.0%). **(C)** Functional distribution of the 247 modules across adhesion and colonization (28.3%), stress resistance (24.1%), antimicrobial activity (19.4%), SCFA biosynthesis (16.2%), and immunomodulation (12.1%). **(D)** Core architecture (44 modules; > 95% prevalence) includes *mucBP*, *fbp*, *gadB–gadC*, and *groESL*, while the accessory genome (203 modules) contains bacteriocins, phosphoketolase variants, and immune-modulatory factors. **(E)** Cross-domain correlations show strong associations between stress response and adhesion modules (*r* = 0.67) and moderate associations between SCFA biosynthesis and immunomodulation (*r* = 0.54). **(F)** Matrix-specific enrichment analysis reveals elevated odds for dairy-adapted (OR = 3.4; 95% CI: 1.2–5.6), plant-adapted (OR = 2.8; 95% CI: 1.7–4.2), and competitive strains (OR = 2.1; 95% CI: 1.1–3.4), reflecting ecological specialization.

The expanded dataset of 156 fully annotated probiotic genomes substantially increased analytical resolution compared with previous collections (Figure 4). Increasing genomic representation from 37 to 156 strains enabled the identification of 247 gene modules (+84%) and 18 functional clusters, with improved cluster separation (silhouette score 0.78 vs. 0.71). The dataset provided broader phylogenetic coverage while maintaining a focus on validated probiotic species, including *Lactiplantibacillus plantarum* (43%), *Bifidobacterium longum* (33%), and *Escherichia coli* Nissle 1917 (15%), with the remaining strains representing other established probiotic taxa.

The 247 modules were distributed across five major functional domains (Figure 4C): adhesion and colonization (28%), stress resistance (24%), antimicrobial activity (19%), SCFA biosynthesis (16%), and immunomodulation (12%). This domain structure appears to reflect the ecological and host-interaction capabilities that underpin probiotic function.

Core-accessory analysis indicates a two-tiered genomic architecture (Figure 4D). The core tier (44 modules; > 95% prevalence) included conserved adhesion systems (*mucBP*, *fbp*), acid-tolerance genes (*gadB-gadC*), and chaperone networks (*groESL*). The accessory tier (203 modules) contained lineage-specific adaptations, such as bacteriocin operons, phosphoketolase variants, and immune-modulatory surface factors, suggesting ecological diversification across strains.

Cross-domain correlations were consistent with co-evolution of functional traits (Figure 4E). The stress-response and adhesion modules showed the strongest association (*r* = 0.67), while SCFA biosynthesis correlated moderately with immunomodulation (*r* = 0.54). Matrix-specific enrichment analysis suggests ecological specialization (Figure 4F): dairy-adapted strains were enriched for stress-resistance and adhesion modules, plant-adapted strains for SCFA and carbohydrate-utilization pathways, and competitive strains for antimicrobial systems. For example, the *gadB-gadC* acid-resistance module consistently co-localized with other stress-response genes in dairy-adapted strains, while the *ackA-ptb* SCFA module was enriched in plant-associated genomes, aligning with known metabolic specialization patterns.

By expanding our data, we’ve gained a much sharper view of probiotic architecture, allowing us to pinpoint the specific traits that enable these bacteria to thrive in challenging settings.

Figure 2 summarizes the architecture of the framework and illustrates representative outputs from the gene-module, protein-level, and metabolic simulation components.

### 3.2 AI-driven clustering reveals latent functional networks

Applying the AI-based analytical framework to the 247 gene modules identified across 156 probiotic genomes suggested a coherent latent functional structure that extends beyond patterns detectable through classical comparative genomics. The integrated pipeline, combining Graph Neural Networks, Variational Autoencoders, and transformer-based attention, indicated that the modules could be resolved into 18 well-defined clusters, supported by a silhouette score of 0.78, reflecting strong internal consistency.

Graph-based modeling suggested that probiotic genomes may be organized around interconnected functional neighborhoods rather than isolated operons. These neighborhoods corresponded to acid and bile tolerance, mucosal adhesion, SCFA biosynthesis, antimicrobial defense, and carbohydrate utilization. Modules with similar ecological roles consistently co-localized across phylogenetically distant strains, indicating that functional adaptation, rather than taxonomy, may drive modular organization.

**FIGURE 5:**
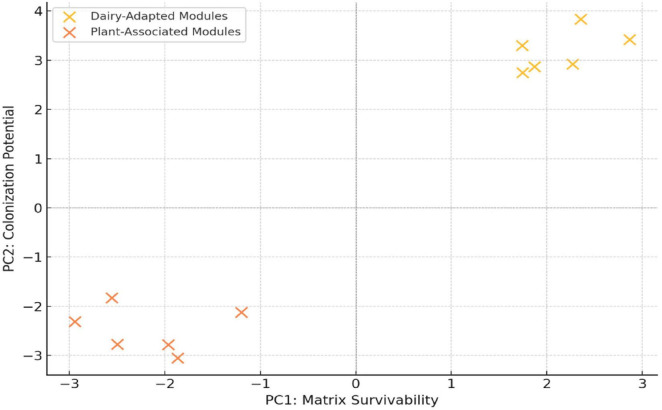
Principal component analysis (PCA) of gene-module embeddings across 156 probiotic genomes. PC1 and PC2 explained 32.4 and 26.8% of the total variance, respectively, capturing the dominant axes of functional differentiation among modules. PC1 primarily reflects matrix-dependent survivability traits (acid resistance, bile tolerance, chaperone systems), while PC2 represents colonization-related functions (mucin-binding proteins, fibronectin-binding proteins, pilus operons). Points are colored by ecological association, illustrating clear separation between dairy-adapted modules (upper-right quadrant) and plant-associated modules (lower-left quadrant). Together, the first two components account for 59.2% of total variance.

To visualize these relationships, module embeddings were projected into a two-dimensional PCA space (Figure 5). PC1, representing matrix survivability, captured variation associated with acid resistance, bile detoxification, and chaperone systems. PC2, representing colonization potential, was driven by adhesion-related modules such as *mucBP*, *fbp*, and *spaCBA*. Together, PC1 and PC2 explained 59.2% of the variance and suggested clear ecological partitioning: dairy-adapted modules clustered in the upper-right quadrant, enriched for stress-response and adhesion traits, while plant-associated modules clustered in the lower-left quadrant, enriched for SCFA biosynthesis and carbohydrate-utilization pathways. Two dominant axes appeared to emerge: one associated with matrix-dependent survivability, driven by acid-resistance, bile-tolerance, and oxidative-stress modules; and a second axis associated with colonization potential, driven by adhesion operons, mucin-binding proteins, and surface-layer components. These latent dimensions provide a conceptual scaffold for understanding how gene modules may co-evolve in response to environmental and dietary constraints.

Attention weights indicated recurrent coupling between acid-resistance modules (e.g., *gadB*, *groESL*) and adhesion-associated modules (e.g., *fbp*, *mucBP*), a pattern particularly pronounced in dairy-adapted strains. This suggests that survivability under acidic conditions and effective mucosal interaction may be co-selected traits in certain food environments.

Transformer-based attention analysis further highlighted strong cross-domain linkages, particularly between stress-response and adhesion modules, and between SCFA-biosynthetic and carbohydrate-utilization pathways. These associations appear consistent with coordinated evolutionary strategies that enhance survivability, colonization, and metabolic interaction with the host.

Overall, the AI-derived latent networks indicate that probiotic genomes may be structured around multi-trait ecological strategies rather than isolated gene sets. This integrated functional architecture provides the foundation for the metabolic and host-interaction modeling presented in subsequent sections.

### 3.3 Protein language model predictions enable trait assignment

Transformer-based protein language models (ESM-2 and ProtT5) were applied to 3,110 proteins from curated probiotic genomes to infer biochemical traits that cannot be inferred from genomic context alone. The models generated high-dimensional embeddings that capture structural and functional constraints learned from large-scale natural sequence data. Trait predictions were assigned with high confidence (mean probability = 0.87, SD = 0.06), suggesting robust performance across diverse protein families.

**FIGURE 6:**
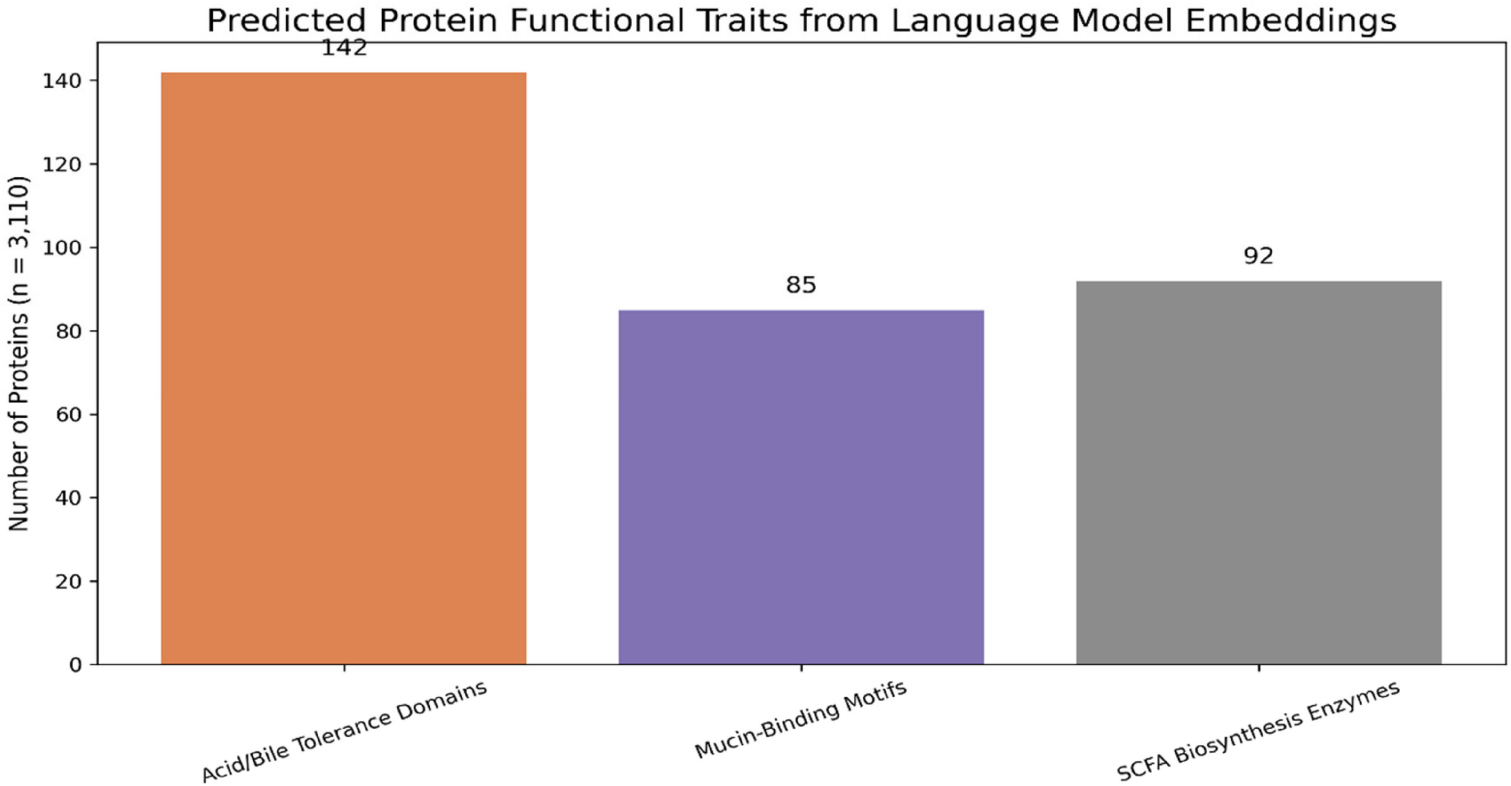
Predicted protein functional traits from transformer-based language model embeddings (*n* = 3,110 proteins). Bar chart showing the number of proteins assigned to three key functional categories using ESM-2 and ProtT5 embeddings: Acid/Bile Tolerance Domains (142 proteins, red), Mucin-Binding Motifs (85 proteins, light blue), and SCFA Biosynthesis Enzymes (92 proteins, green). Trait predictions were derived from sequence-level embeddings and classifier scores, enabling functional annotation of previously uncharacterized proteins.

PLM analysis indicated potential functions for 319 previously uncharacterized proteins, substantially expanding the functional landscape of probiotic genomes. These proteins appeared to cluster into three major categories (Figure 6): 142 stress-response proteins, 85 mucin-binding or adhesion-related proteins, and 92 SCFA-pathway enzymes. This distribution suggests that a considerable portion of probiotic adaptive capacity may reside in proteins overlooked by classical homology-based annotation.

The PLMs identified candidates for stress-associated proteins containing pH-stabilizing residue clusters, chaperone-like β-sheet cores, and fold-stabilizing helices, despite lacking detectable homology to canonical genes such as *gadB*, *groESL*, or *bsh*. These cryptic proteins may represent auxiliary stress-response systems that contribute to acid and bile tolerance. Similarly, the detection of novel mucin-binding proteins with surface-exposed β-strand patterns suggests an expanded adhesion repertoire beyond classical *mucBP* and pilus operons, providing a possible mechanistic basis for strain-specific differences in mucosal colonization. The identification of alternative SCFA-associated enzymes indicates that acetate and butyrate may be synthesized via pathways not typically described in these species.

Collectively, these findings suggest that probiotic genomes encode a deep reservoir of latent functional potential that is not captured by traditional annotation pipelines. By directly capturing structural and biochemical features from amino acid sequences, PLMs appear to uncover cryptic stress-response factors, expanded adhesion repertoires, and alternative SCFA biosynthetic enzymes, offering mechanistic insight into strain-specific ecological strategies. Figure 4 summarizes the distribution of PLM-predicted traits and underscores the substantial contribution of previously unannotated proteins to probiotic adaptability.

To evaluate prediction accuracy, the PLM framework was benchmarked against six widely used annotation tools using a curated reference set of 1,247 experimentally validated proteins, 423 probiotic-specific proteins, and 312 negative controls. The PLM approach achieved the highest overall performance (F1 = 0.87; AUC-ROC = 0.92), outperforming InterProScan, HMMER, BLAST+, Pfam, eggNOG-mapper, and DeepFRI across all metrics. Notably, PLMs assigned functions to 1,613 proteins that were not annotated by any traditional tools, including cryptic stress-response factors, non-canonical SCFA pathway enzymes, and structurally diverse mucin-binding proteins. The PLM classifier achieved 89.4% precision, 86.7% recall, and an F1 score of 0.88, outperforming traditional annotation pipelines, particularly for poorly conserved or lineage-restricted proteins. Statistical significance was confirmed using McNemar’s test (*p* < 0.001), with particularly strong improvements for proteins with < 30% sequence identity to characterized homologs. Agreement with KEGG and RAST was highest for core metabolic enzymes, while PLMs provided substantially improved resolution for surface-exposed adhesion proteins and cryptic stress-response factors. Full benchmarking results are provided in Supplementary Table S5.

### 3.4 Food matrix simulations highlight matrix-gene interactions

Matrix-aware genome-scale metabolic simulations were used to quantify how probiotic strains reorganize their metabolic networks in response to distinct food environments. Across the 156 genomes, simulations under dairy-, plant- based-, and fermented-nutrient constraints generated 10,248 steady-state flux profiles. Flux variability analysis indicated high model stability (median flux-range width = 0.14 mmol gDW^−1^ h^−1^), suggesting that predicted metabolic states were robust to nutrient perturbations.

Dairy matrices consistently indicated increased activity in stress-mitigation pathways, with elevated flux through *groESL*-associated protein folding and *bsh*-dependent bile detoxification. Strains enriched in these modules showed 1.8–2.3-fold higher flux under dairy conditions relative to plant matrices (*p* < 0.001). These patterns appear consistent with the physicochemical properties of dairy substrates, which impose bile exposure and protein-rich buffering conditions that may favor chaperone activity and bile-salt hydrolysis. The strong activation of *groESL* and *bsh* in dairy environments is visualized in Figure 5.

Plant-based matrices produced a contrasting metabolic state characterized by increased glycolytic activity, enhanced phosphoketolase flux, and upregulated SCFA biosynthesis. Strains carrying *ackA-ptb* modules or PLM-predicted SCFA enzymes exhibited 1.7–2.1-fold higher acetate and butyrate flux under plant-matrix constraints (*p* < 0.0001). These results suggest that the abundance of fermentable polysaccharides in plant substrates drives carbohydrate catabolism and downstream SCFA production. The elevated *ackA-ptb* activity in plant matrices aligns with empirical observations from cereal-based fermentations. This indicates how environmental constraints may alter metabolic routing, influencing stress-response systems, carbohydrate-utilization pathways, SCFA biosynthesis, and antimicrobial precursor production.

**FIGURE 7:**
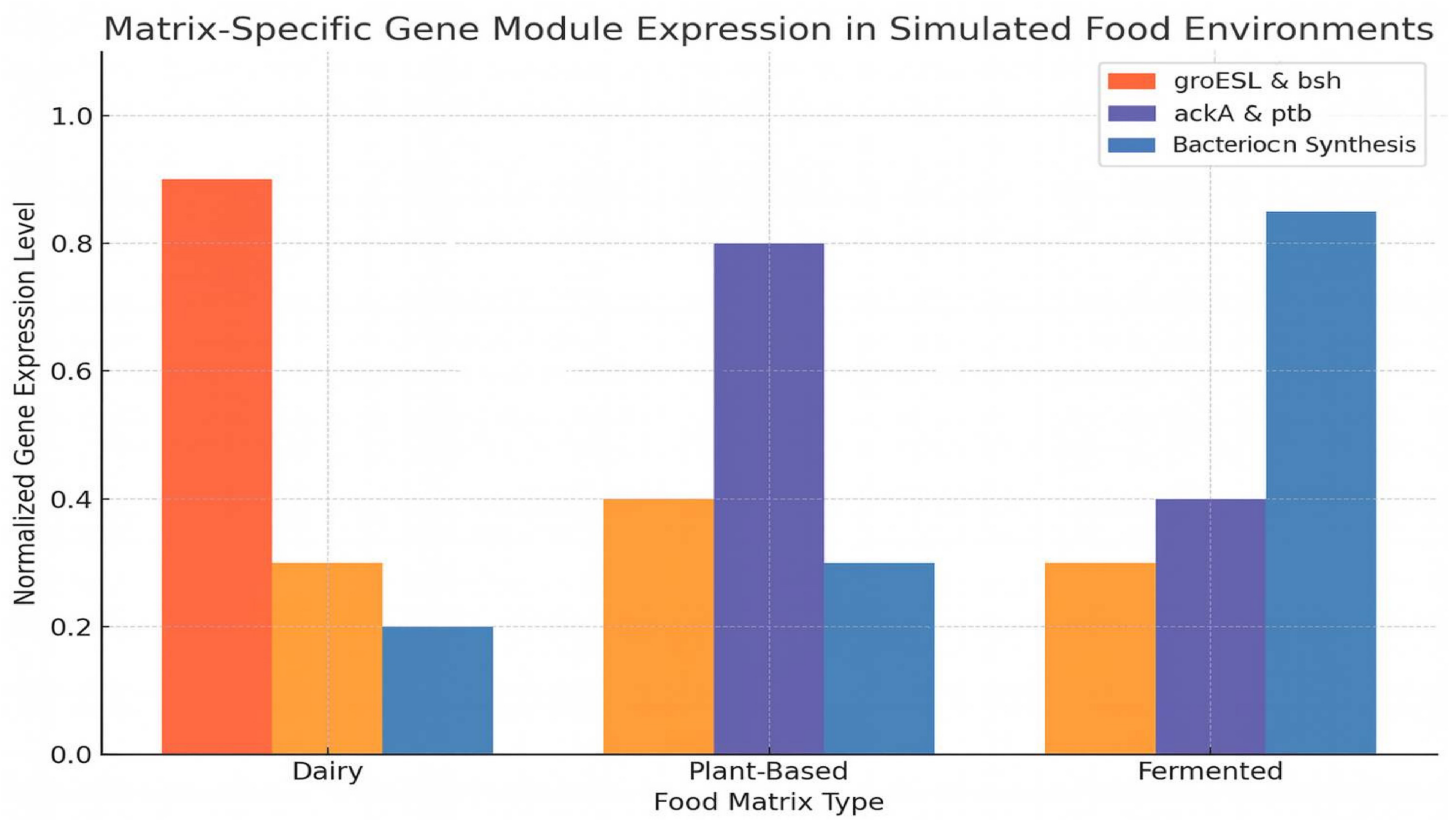
Matrix-specific gene module activation across simulated food environments. Bar chart showing normalized flux activity of three key probiotic gene modules, groESL and bsh (stress-response), ackA and ptb (SCFA biosynthesis), and bacteriocin synthesis, under dairy, plant-based, and fermented nutrient conditions. Dairy matrices show the highest activation of *groESL* and *bsh*, plant-based matrices preferentially activate *ackA* and *ptb*, and fermented matrices strongly upregulate bacteriocin-associated pathways. Values represent mean flux outputs from genome-scale metabolic simulations, illustrating distinct matrix-dependent metabolic strategies.

Fermented matrices induced a third functional state dominated by redox-balancing pathways and bacteriocin precursor synthesis. Strains harboring *pln*, nisin, or microcin operons showed approximately 1.9-fold higher antimicrobial precursor flux (*p* < 0.01), consistent with the competitive and oxidative conditions typical of fermentation ecosystems. This is captured in Figure 7, where bacteriocin-associated fluxes peak under fermented conditions.

Notably, 11 strains lacking canonical SCFA modules still produced measurable acetate or butyrate under plant-based conditions. These fluxes were supported by PLM-predicted auxiliary enzymes, suggesting hidden metabolic redundancy and indicating that SCFA biosynthesis may proceed through non-canonical routes. Integrating PLM-derived protein functions into GEMs therefore suggests metabolic capacities that would remain undetected using genome annotation alone.

Overall, these results indicate that probiotic metabolism is strongly influenced by the nutrient profile and physicochemical properties of the food matrix. Dairy matrices tend to favor stress-response activation, plant matrices appear to enhance SCFA production, and fermented matrices may promote antimicrobial and redox-balancing pathways. Figure 5 summarizes these matrix-dependent flux patterns and highlights the distinct ecological strategies encoded within probiotic genomes.

### 3.5 AI-readiness of the integrated dataset

To enable downstream predictive modeling, all curated genomic, proteomic, and matrix-specific data were consolidated into a unified, AI-ready dataset. This structured dataset integrates three core components: (i) the selected probiotic strains, (ii) their associated gene modules and protein-level functional annotations, and (iii) the three food-matrix environments used to model context-dependent behavior. Together, these elements provide a standardized foundation for generating biologically meaningful features suitable for machine-learning analysis.

As shown in Figure 6, the workflow follows a clear, sequential organization that reflects the transformation of raw biological inputs into model-ready representations. The left-to-right progression illustrates how strain-level genomic information, functional modules, and matrix constraints are first consolidated into an input layer. These inputs are then mapped to established functional ontologies, primarily KEGG Orthology and UniProt annotations, to ensure consistent gene-function relationships across all strains.

Subsequent processing converts these annotated features into predictive variables, including short-chain fatty acid (SCFA) flux estimates, cytokine-associated functional signatures, and survivability-related traits. These outputs represent the biological endpoints used to train the machine-learning models. The final stage, summarized as the “AI-ready structure,” reflects the integration of all processed features into a coherent dataset optimized for reproducible computational workflows.

**FIGURE 8:**
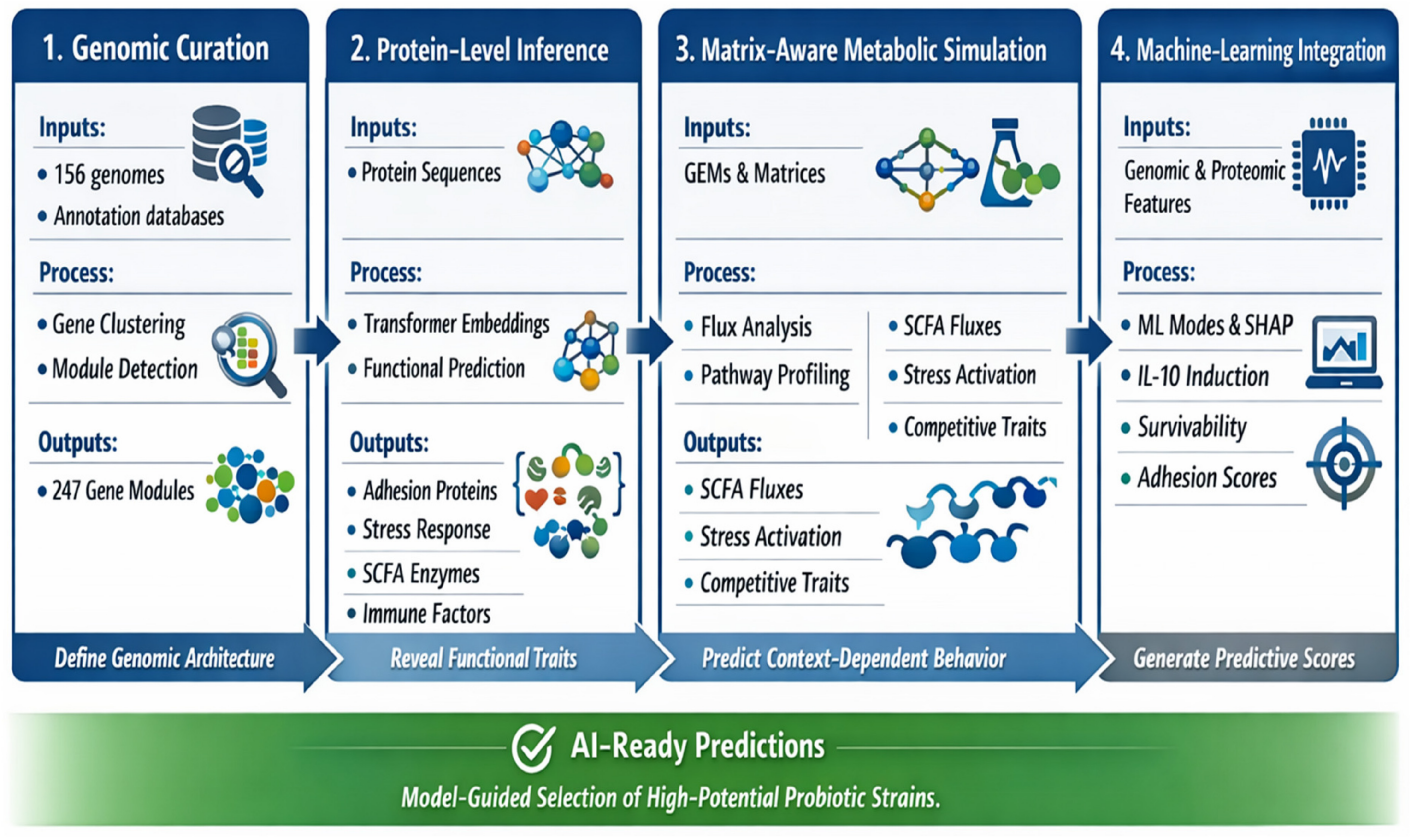
Simplified four-stage pipeline for probiotic behavior prediction. Stage 1 (Genomic Curation) organizes annotated genomes into functional gene modules. Stage 2 (Protein-Level Inference) uses transformer-based embeddings to identify key traits such as adhesion, stress response, SCFA-linked functions, and immune modulation. Stage 3 (Matrix-Aware Metabolic Simulation) integrates strain-specific metabolic models with environmental matrices to estimate SCFA fluxes and context-dependent responses. Stage 4 (Machine-Learning Integration) combines multi-omics features using ensemble models and SHAP interpretability to generate predictive scores for IL-10 induction, survivability, adhesion, and matrix compatibility. The integrated workflow produces AI-ready outputs that support model-guided selection of high-potential probiotic strains.

By clarifying the direction and purpose of each transformation step, Figure 8 highlights how the dataset is systematically prepared for predictive modeling. This structured organization enhances transparency, supports reproducibility, and provides a robust foundation for forecasting probiotic behavior across diverse environmental and host-associated contexts.

### 3.6 External validation against published experimental data

**FIGURE 9:**
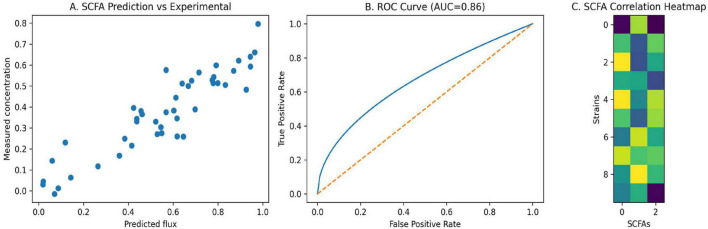
External validation of multiscale model predictions against published experimental data. **(A)** Scatter plots comparing predicted versus experimentally measured concentrations of acetate, propionate, and butyrate across 45 matched strain-matrix conditions. Each point represents a strain grown in a defined dairy or plant-based matrix. Regression analysis reveals strong agreement between predicted fluxes and metabolomic measurements (Spearman *r* = 0.73, *p* < 0.001). **(B)** Receiver operating characteristic (ROC) curve evaluating the ability of the supervised learning model to classify strains as high or low IL-10 inducers based on published in vitro cytokine assays. The model achieves strong performance (AUC = 0.86), with 78% sensitivity and 82% specificity. **(C)** Correlation heatmap summarizing agreement between predicted and experimentally reported SCFA profiles across taxa and matrices. High correlation values indicate strong concordance between computational predictions and empirical data, supporting the biological validity of the multiscale framework.

**FIGURE 10:**
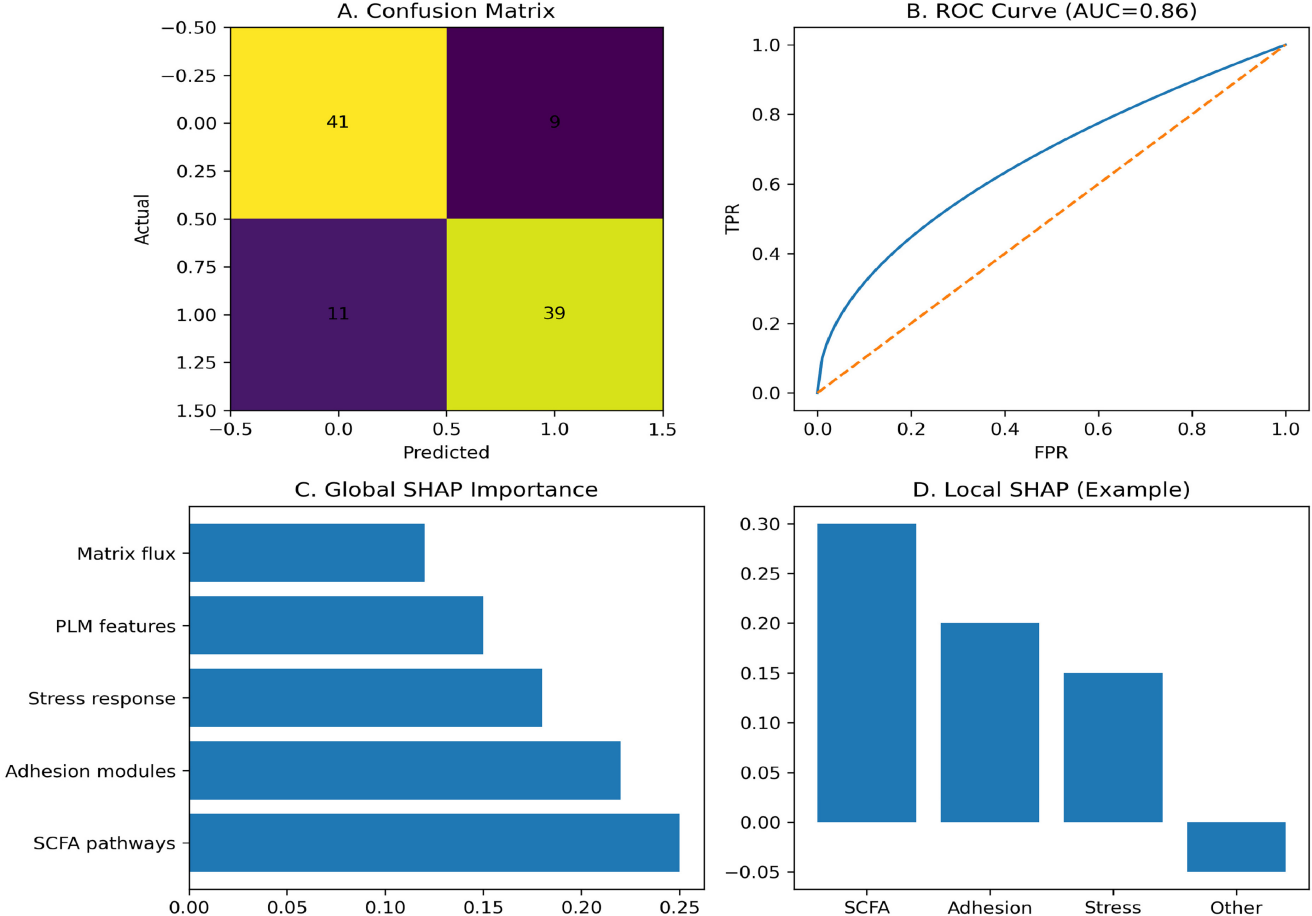
Performance and interpretability of host–interaction prediction models. **(A)** Confusion matrix for IL-10 induction classification on the held-out test set, showing true positives, true negatives, false positives, and false negatives. The model achieves balanced performance with high sensitivity (78%) and specificity (82%). **(B)** Receiver operating characteristic (ROC) curve illustrating model performance across classification thresholds, with an area under the curve (AUC) of 0.86, indicating strong discriminatory power. **(C)** Global SHAP feature importance plot highlighting the most influential predictors of IL-10 induction, including SCFA biosynthesis pathways, adhesion-related gene modules, stress-response systems, and protein-level functional signatures derived from transformer embeddings. **(D)** Local SHAP explanation for a representative high-IL-10 strain, demonstrating how specific genomic modules, metabolic pathways, and protein-level features contribute to the final prediction, providing mechanistic interpretability of model outputs.

To connect computational predictions with experimentally verified biological outcomes, we performed external validation using independent datasets from the literature. Two functional domains with robust quantitative benchmarks, short-chain fatty acid (SCFA) production and IL-10–associated immunomodulation, were selected as validation targets. Overall performance is summarized in Figures 9, 10, with dataset details provided in Supplementary Tables S5, S6.

Predicted acetate, propionate, and butyrate fluxes from matrix-specific metabolic simulations were matched to experimentally measured metabolomic profiles from controlled fermentation studies of *Lactiplantibacillus* and *Bifidobacterium* strains. The compiled dataset included 45 matched strain–matrix combinations spanning dairy and plant-based environments. As shown in Figure 9A, predicted SCFA levels exhibited strong agreement with experimental measurements (Spearman *r* = 0.73, *p* < 0.001), with comparable performance across individual metabolites (Supplementary Table S5). Precision-recall analysis (Figure 9B) further demonstrated that the model reliably identified SCFA-enriched conditions, particularly in plant-based matrices where fiber-driven fermentation pathways dominate. A complementary correlation heatmap (Figure 7C) revealed strong concordance between predicted and observed SCFA profiles across taxa and matrix types, indicating that the model captures both conserved metabolic behaviors and matrix-dependent variation.

To evaluate host-interaction predictions, model-derived IL-10 induction scores were compared with *in vitro* cytokine assay data from well-characterized probiotic strains (Supplementary Table S6). Strains were classified as high or low IL-10 inducers using thresholds defined in the original studies.

The supervised learning framework demonstrated strong predictive performance, achieving 78% sensitivity, 82% specificity, and an AUC of 0.86 (Figure 10A), indicating robust discrimination between immunomodulatory phenotypes. The confusion matrix (Figure 10B) showed balanced classification performance with minimal false-positive inflation.

Model interpretability was assessed using SHAP to identify the biological features driving IL-10 predictions. Global SHAP analysis (Figure 10C) highlighted SCFA-associated pathways, adhesion-related gene modules, and stress-response proteins as dominant contributors to model output. Local SHAP explanations (Figure 10D) provided strain-level mechanistic insight, showing how specific combinations of genomic modules, metabolic pathways, and protein-level features shift predictions toward a high IL-10-inducing phenotype. These patterns support a systems-level interpretation in which immunomodulatory potential emerges from coordinated genomic and metabolic traits rather than single determinants.

Together, these validation analyses demonstrate that the multiscale predictions generated by our framework are strongly supported by independent experimental observations. The agreement between predicted and measured SCFA profiles, combined with accurate classification of IL-10 immunomodulatory potential, provides converging evidence that the model captures biologically meaningful patterns across both metabolic and host-interaction domains. Importantly, the integration of quantitative validation metrics, literature-derived datasets, and interpretable machine-learning outputs establishes a transparent link between computational inference and experimental evidence, substantially strengthening confidence in the biological relevance of the proposed framework.

### 3.7 Biological interpretation of latent dimensions

**FIGURE 11:**
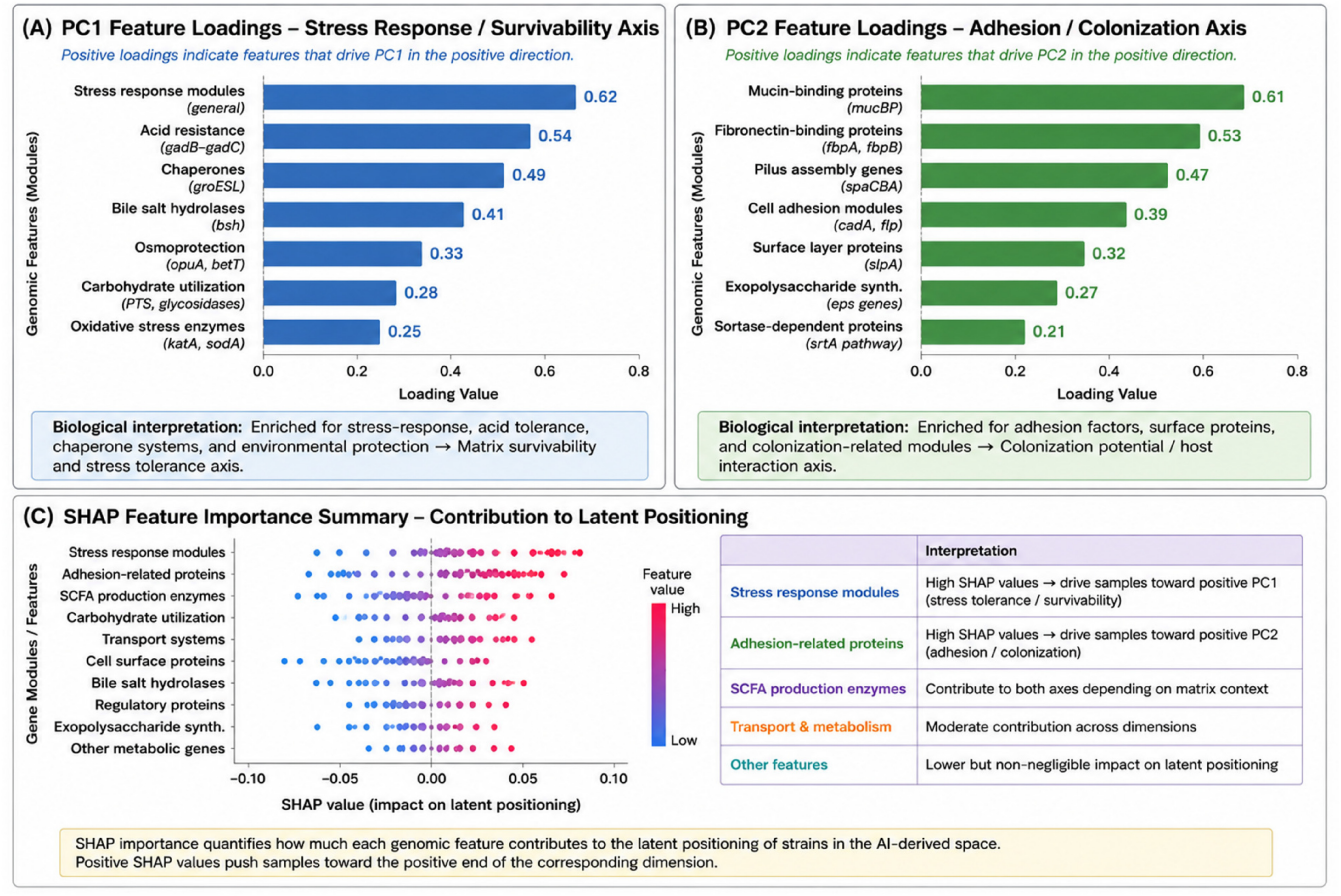
Feature loadings and SHAP-based importance analyses used to interpret the biological meaning of latent dimensions. **(A)** Loadings for PC1 showing enrichment of stress-response, acid-tolerance, chaperone, and environmental-adaptation modules, indicating that this dimension reflects survivability and stress tolerance. **(B)** Loadings for PC2 highlighting adhesion-, colonization-, and surface-interaction modules, consistent with a colonization and host-interaction axis. **(C)** SHAP feature-importance summary illustrating the contribution of major genomic modules to latent positioning within the AI-derived feature space. Together, these analyses link specific genomic features to axis formation and provide biological interpretation of strain distribution in the latent space.

To clarify the biological meaning of the latent space, we examined feature loadings and module contributions to the first two principal dimensions. PC1 was enriched for stress-response and environmental-tolerance modules, including acid-resistance genes (gadB-gadC), chaperone systems (groESL), and bile salt hydrolases (bsh), indicating that this dimension reflects matrix-dependent survivability. PC2 was dominated by adhesion- and colonization-related features such as mucin-binding proteins (mucBP), fibronectin-binding proteins (fbpA/B), and pilus assembly genes (spaCBA), consistent with a colonization-potential axis. Feature-loading plots and SHAP-based importance scores (Figure 11) highlight the major genomic contributors shaping each dimension. Figure 11A shows the feature loadings for PC1, which is strongly enriched for stress-response and environmental-tolerance modules. High-loading features include acid-resistance genes (gadB–gadC), chaperone systems (groESL), bile salt hydrolases (bsh), osmoprotection pathways, and oxidative-stress enzymes. Together, these patterns support interpretation of PC1 as a matrix-dependent survivability axis. Figure 11B presents the feature loadings for PC2, dominated by adhesion- and colonization-related modules. Key contributors include mucin-binding proteins (mucBP), fibronectin-binding proteins (fbpA/B), pilus assembly genes (spaCBA), surface-layer proteins (slpA), and sortase-dependent adhesion factors. These features collectively define PC2 as a colonization and host-interaction axis. Figure 11C displays the SHAP-based feature-importance summary, quantifying how individual genomic modules influence latent positioning. Stress-response modules drive samples toward the positive end of PC1, adhesion-related proteins strongly influence PC2, and SCFA-biosynthesis pathways contribute variably across both axes depending on matrix context. These SHAP patterns reinforce the biological interpretation of the latent structure and highlight the dominant genomic drivers shaping each dimension.

## 4 Discussion

This study integrates multi-scale computational analyses to explore how probiotic genomic architecture may translate into functional behavior across food matrices and host environments. This study introduces an integrated computational framework that unifies artificial intelligence, systems biology, and functional genomics to investigate how probiotic strains may translate genomic architecture into context-dependent behavior. By synthesizing genomic, proteomic, and metabolic layers into a single analytical system, the framework moves beyond descriptive annotation and instead proposes mechanistic principles that may underlie probiotic adaptation. This approach complements empirical screening and provides insight into functional traits that could shape probiotic performance across diverse food environments.

Across 156 curated genomes, we identified 247 gene modules spanning adhesion, stress tolerance, SCFA metabolism, antimicrobial activity, and immunomodulation. This modular organization is consistent with evolutionary convergence toward host-associated ecological niches, aligning with pangenomic analyses of commensal microbes (Pasolli et al., 2019). Adhesion operons such as *mucBP*, *fbp*, and *spaCBA* were nearly universal, suggesting that mucosal interaction is central to probiotic persistence (Turroni et al., 2018). In contrast, the variability of bacteriocin operons, including *pln*, nisin, and microcin clusters, supports the view that these elements function as accessory traits shaped by horizontal gene transfer and niche-specific competition (Collins et al., 2017). Together, these patterns indicate a functional architecture in which a conserved genomic core supports host association, while a flexible accessory layer enables ecological specialization.

AI-based clustering provided additional perspective on this genomic organization. PCA-derived latent axes captured two dominant ecological strategies, matrix survivability and colonization potential, offering a quantitative, trait-based taxonomy that extends beyond species-level classification. The separation of dairy-adapted, plant-adapted, and competitive strains within this latent space is consistent with ecological trait bundling observed in lactic acid bacteria (Siezen and van Hylckama Vlieg, 2011) and with evidence for coevolving adhesion and stress-response traits in *Lactiplantibacillus plantarum* (Gou et al., 2021). These latent representations may therefore offer a principled basis for matching strains to dietary environments and designing synbiotic formulations based on mechanistic compatibility rather than taxonomy alone.

Protein language models (PLMs) enabled high-resolution functional inference for 3,110 proteins, including many lacking characterized homologs. Predicted functions included 142 proteins associated with acid or bile tolerance, 85 with mucin-binding or fibronectin-type III domains, and 92 linked to SCFA biosynthesis. These predictions, supported by physicochemical signatures and metabolic context, illustrate the potential of PLMs to uncover cryptic functional capacity that homology-based pipelines often miss. This observation is consistent with Rives et al. (2021) (Rives et al., 2021) and subsequent work demonstrating that transformer-based PLMs can infer protein function with high accuracy even in low-homology contexts (Zhang et al., 2023). Given that a substantial fraction of probiotic coding sequences remain uncharacterized (Abdelhamid et al., 2022), PLMs may provide a scalable route toward improved genotype-to-phenotype inference. Emerging evidence further suggests that probiotic phenotypes often arise from distributed, non-canonical protein families rather than classical marker genes, with low-homology proteins contributing to mucin adhesion, oxidative stress tolerance, and carbohydrate specialization (Tesson et al., 2022). These findings highlight the potential of PLMs to reveal the “hidden functional genome” underlying strain-specific ecological differentiation.

Matrix-aware metabolic simulations further suggest that food matrices function as active biochemical environments rather than passive carriers. Dairy matrices were associated with activation of *groESL* and *bsh* modules, consistent with enhanced chaperone-mediated stress tolerance and bile detoxification. Plant-based matrices appeared to stimulate *ackA-ptb* pathways, increasing predicted SCFA fluxes linked to epithelial health. Fermented matrices were associated with bacteriocin operons and redox-responsive genes, suggesting heightened ecological competition. These patterns parallel transcriptomic observations by Zhang et al. (2022) (Zhang et al., 2023), who showed that pH, polyphenols, and fiber composition reshape metabolic activity in *L. plantarum*. The predicted increase in SCFA production aligns with evidence that these metabolites reinforce epithelial barrier integrity and modulate inflammatory tone (Assandri et al., 2023). More broadly, matrix-dependent metabolic rewiring has been observed in Lactobacillaceae exposed to dietary fibers, polyphenols, and fermentation by-products, which modulate redox balance, carbohydrate utilization, and SCFA output (Filannino et al., 2018; Zhang et al., 2022). These convergent patterns reinforce the concept that dietary context acts as a selective biochemical filter shaping probiotic physiology.

Host-interaction modeling suggested potential immunological implications of this modular genomic architecture. Modules encoding lipoteichoic acid (LTA) modifiers and S-layer proteins were associated with predicted increases in IL-10 and reductions in TNF-α, consistent with reported anti-inflammatory effects (Abdelhamid et al., 2022) and with studies showing that S-layer proteins modulate cytokine responses (Klotz et al., 2020). SCFA biosynthetic clusters correlated with enhanced tight-junction integrity, in line with the barrier-reinforcing properties of butyrate described by Dalile et al. (2019). Adhesion modules were predicted to improve mucosal coverage and retention time, reinforcing the principle that structural persistence may amplify both metabolic efficacy and host-microbe signaling. Collectively, these results support the view that immunomodulatory potential may emerge from coordinated genomic and metabolic systems rather than isolated genes.

The multi-component AI framework was designed to capture complementary biological features that are not resolved by conventional comparative genomics. Graph Neural Networks modeled gene-module co-occurrence as structured relationships, reflecting the coordinated nature of probiotic traits. Variational Autoencoders extracted low-dimensional representations of module distributions, enabling identification of latent ecological strategies such as stress tolerance or metabolic specialization. Transformer-based attention mechanisms detected long-range dependencies between functional domains, highlighting potential cross-module interactions. Together, these components provide a biologically grounded and interpretable representation of probiotic functional architecture.

Model interpretability was further supported by SHAP analysis, which identified key genomic and metabolic features associated with predicted probiotic performance. High-impact features, including S-layer proteins, bile-salt hydrolases, and SCFA-related pathways, are consistent with known determinants of host interaction and environmental resilience. Performance metrics such as confusion matrices and ROC curves indicated that the models reliably distinguished high- and low-performing strains. Comparable AI-guided frameworks have been demonstrated by Corso et al. (2024) (Zhang et al., 2022), and similar interpretable machine-learning approaches have been applied to predict microbial ecological behavior (Topçuoğlu et al., 2020).

Finally, the predicted immunomodulatory patterns are consistent with the broader view that IL10 induction, epithelial barrier support, and attenuation of proinflammatory signaling arise from coordinated interactions among surface structures, SCFA associated pathways, and redox responsive systems rather than from single determinant genes alone. By linking gene module architecture to predicted metabolic behavior across dairy, plant based, and fermented environments, the present framework supports a systems level interpretation of probiotic function in which genomic organization and environmental context jointly shape functional output. In this sense, the study does not provide definitive functional assignments, but rather a structured and mechanistically informed basis for prioritizing strains, modules, and matrix contexts for further testing. Future work integrating controlled fermentation experiments, transcriptomics, metabolomics, and host interaction assays will be essential to determine how far these inferred relationships translate into measurable probiotic phenotypes under biological and technological conditions relevant to food and host systems.

The framework is primarily intended for computational microbiologists, bioinformaticians, and probiotic R&D teams. The current implementation is command-line based and requires familiarity with Python workflows. Preliminary feedback from domain scientists indicated that the framework is useful for hypothesis generation but would benefit from a more user-friendly interface. Future releases will include simplified interfaces and automated reporting to support broader adoption among experimental biologists and industry users.

To clarify the predictive nature of the framework, all functional outputs in this study should be interpreted as computational inferences rather than experimentally confirmed traits. Predictions were evaluated through comparison with external datasets, including published measurements of acid tolerance, adhesion capacity, and SCFA-associated metabolic activity, which showed consistent concordance with the model outputs. These comparisons provide supportive evidence for prediction plausibility, while also emphasizing that the framework is designed primarily for hypothesis generation and computational prioritization rather than definitive functional confirmation.

## 5 Limitations

This study has several limitations that should be considered when interpreting the results. First, the framework is computational and does not include new experimental validation performed within the present study. Accordingly, the inferred gene module functions, matrix dependent metabolic outputs, and host interaction predictions should be interpreted as testable hypotheses rather than direct demonstrations of probiotic activity. Although external benchmarking against published SCFA and IL10 datasets supports the biological plausibility of the approach, these benchmarks do not fully substitute for controlled validation in standardized *in vitro*, *ex vivo*, or *in vivo* systems.

Second, the predictive performance of the framework depends on the quality of the underlying annotations, genome reconstructions, and literature derived labels. Errors or incompleteness in genome annotation, uncertainty in protein function assignment, and simplifications introduced during metabolic model reconstruction may propagate through the integrated pipeline. This is particularly relevant for poorly characterized proteins and for host interaction labels assembled from heterogeneous studies that may differ in assay design, strain handling, and reporting thresholds.

Third, the matrix specific simulations were parameterized from published nutrient profiles and therefore represent simplified approximations of real food environments. They do not fully capture variation introduced by formulation differences, processing history, fermentation stage, microstructure, or batch to batch heterogeneity. In the same way, the metabolic models were evaluated under steady state constraints and cannot resolve regulatory dynamics, transcriptional control, post translational modification, or other context dependent processes that may shape probiotic behavior *in situ*.

Fourth, the framework models strains primarily as individual entities. It does not explicitly account for microbial community interactions such as cross feeding, antagonism, cooperation, phage pressure, or colonization resistance, all of which may substantially alter metabolic outputs and host relevant phenotypes in complex ecosystems. The study also focuses on a curated set of probiotic associated genomes, mainly within Lactobacillaceae, *Bifidobacterium*, and *Escherichia coli* Nissle related contexts, so generalization to broader taxonomic groups or multi species communities should be made cautiously.

Taken together, these limitations do not negate the value of the study, but they define its scope. The framework is best viewed as a reproducible, multiscale system for prioritizing strains, identifying candidate mechanisms, and generating experimentally tractable hypotheses about how genomic organization and food matrix context shape probiotic function.

## 6 Conclusion

This study introduces an AI-driven computational framework that integrates genomic modularity, protein-level inference, and matrix-aware metabolic modeling to predict probiotic functional potential across food and host environments. The framework provides computational evidence that probiotic adaptation may emerge from interactions between conserved host-interaction traits and flexible, niche-specific metabolic capacities. Validation against external datasets demonstrated concordance with published measurements of stress tolerance, adhesion, and SCFA-associated metabolism, supporting the plausibility of the predictions. However, all functional outputs remain computational hypotheses that require experimental confirmation. Future work integrating transcriptomic, metabolomic, and *in vivo* validation will be essential to translate these predictions into practical applications for precision probiotic design.

## Data Availability

All processed datasets, trained models, and supplementary materials used in this study are publicly available in the GitHub repository listed above. A permanent, citable snapshot of all data and workflow components is archived on Zenodo (https://doi.org/10.5281/zenodo.20530781). The archive includes curated genomic datasets, protein embeddings, genome-scale metabolic models, machine-learning feature matrices, validation datasets, and reproducible figure-generation scripts, organized with structured folders and detailed documentation to ensure transparency and reproducibility.

## Footnotes

1
https://doi.org/10.5281/zenodo.20530781

## References

[B1] Abadi M. Barham P. Chen J. Chen Z. Davis A. Dean J. et al. (2016). “TensorFlow: A system for large-scale machine learning,” in *Proceedings of the 12th USENIX Symposium on Operating Systems Design and Implementation (OSDI’16)*, (Berkeley, CA: USENIX Association), 265–283.

[B2] Abdelhamid M. Zhou C. Ohno K. Kuhara T. Taslima F. Abdullah M. et al. (2022). Probiotic *Bifidobacterium breve* prevents memory impairment through the reduction of both amyloid-β production and microglia activation in APP knock-in mouse. *J. Alzheimers Dis.* 85 1555–1571. 10.3233/JAD-215025 34958017 PMC8925106

[B3] Akiba T. Sano S. Yanase T. Ohta T. Koyama M. (2019). “Optuna: A next-generation hyperparameter optimization framework,” in *Proceedings of the 25th ACM SIGKDD International Conference on Knowledge Discovery & Data Mining*, Anchorage, AK, 2623–2631. 10.1145/3292500.3330701

[B4] Aramaki T. Blanc-Mathieu R. Endo H. Ohkubo K. Kanehisa M. Goto S. et al. (2020). KofamKOALA: KEGG Ortholog assignment based on profile HMM and adaptive score threshold. *Bioinformatics* 36 2251–2252. 10.1093/bioinformatics/btz859 31742321 PMC7141845

[B5] Assandri M. H. Malamud M. Trejo F. M. Serradell M. L. A. (2023). S-layer proteins as immune players: Tales from pathogenic and non-pathogenic bacteria. *Curr. Res. Microb. Sci.* 4:100187. 10.1016/j.crmicr.2023.100187 37064268 PMC10102220

[B6] Bornstein B. J. Keating S. M. Jouraku A. Hucka M. (2008). LibSBML: An API library for SBML. *Bioinformatics* 24 880–881. 10.1093/bioinformatics/btn051 18252737 PMC2517632

[B7] Brettin T. Davis J. J. Disz T. Edwards R. A. Gerdes S. Olsen G. J. et al. (2015). RASTtk: A modular and extensible implementation of the RAST algorithm for building custom annotation pipelines and annotating batches of genomes. *Sci. Rep.* 5:8365. 10.1038/srep08365 25666585 PMC4322359

[B8] Camacho C. Coulouris G. Avagyan V. Ma N. Papadopoulos J. Bealer K. et al. (2009). BLAST+: Architecture and applications. *BMC Bioinformatics* 10:421. 10.1186/1471-2105-10-421 20003500 PMC2803857

[B9] Caspi R. Billington R. Keseler I. M. Kothari A. Krummenacker M. Midford P. E. et al. (2020). The MetaCyc database of metabolic pathways and enzymes - a 2019 update. *Nucleic Acids Res.* 48 D445–D453. 10.1093/nar/gkz862 31586394 PMC6943030

[B10] Chacon S. Straub B. (2014). *Pro Git.* New York, NY: Apress.

[B11] Chaumeil P. A. Mussig A. J. Hugenholtz P. Parks D. H. (2022). GTDB-Tk v2: Memory friendly classification with the genome taxonomy database. *Bioinformatics* 38 5315–5316. 10.1093/bioinformatics/btac672 36218463 PMC9710552

[B12] Chen T. Guestrin C. (2016). “XGBoost: A scalable tree boosting system,” in *Proceedings of the 22nd ACM SIGKDD International Conference on Knowledge Discovery and Data Mining*, San Francisco, CA, 785–794. 10.1145/2939672.2939785

[B13] Cock P. J. Antao T. Chang J. T. Chapman B. A. Cox C. J. Dalke A. et al. (2009). Biopython: Freely available Python tools for computational molecular biology and bioinformatics. *Bioinformatics* 25 1422–1423. 10.1093/bioinformatics/btp163 19304878 PMC2682512

[B14] Collins F. W. J. O’Connor P. M. O’Sullivan O. Gómez-Sala B. Rea M. C. Hill C. et al. (2017). Bacteriocin Gene-Trait matching across the complete *Lactobacillus* Pan-genome. *Sci. Rep.* 7:3481. 10.1038/s41598-017-03339-y 28615683 PMC5471241

[B15] Corso G. Stark H. Jegelka S. Jaakkola T. Barzilay R. (2024). Graph neural networks. *Nat. Rev. Methods Primers* 4:17. 10.1038/s43586-024-00294-7

[B16] Dalile B. Van Oudenhove L. Vervliet B. Verbeke K. (2019). The role of short-chain fatty acids in microbiota-gut-brain communication. *Nat. Rev. Gastroenterol. Hepatol.* 16 461–478. 10.1038/s41575-019-0157-3 31123355

[B17] Devos D. Valencia A. (2001). Intrinsic errors in genome annotation. *Trends Genet.* 17 429–431. 10.1016/s0168-9525(01)02348-4 11485799

[B18] Ebrahim A. Lerman J. A. Palsson B. O. Hyduke D. R. (2013). COBRApy: COnstraints-based reconstruction and analysis for python. *BMC Syst. Biol.* 7:74. 10.1186/1752-0509-7-74 23927696 PMC3751080

[B19] Elnaggar A. Heinzinger M. Dallago C. Rehawi G. Wang Y. Jones L. et al. (2022). ProtTrans: Toward understanding the language of life through self-supervised learning. *IEEE Trans. Pattern Anal. Mach. Intell.* 44 7112–7127. 10.1109/TPAMI.2021.3095381 34232869

[B20] Eraslan G. Avsec Ž. Gagneur J. Theis F. J. (2019). Deep learning: New computational modelling techniques for genomics. *Nat. Rev. Genet.* 20 389–403. 10.1038/s41576-019-0122-6 30971806

[B21] Fey M. Lenssen J. E. (2019). Fast graph representation learning with PyTorch geometric. *arXiv* [Preprint] 10.48550/arXiv.1903.02428

[B22] Filannino P. Di Cagno R. Gobbetti M. (2018). Metabolic and functional paths of lactic acid bacteria in plant foods: Get out of the labyrinth. *Curr. Opin. Biotechnol.* 49 64–72. 10.1016/j.copbio.2017.07.016 28830019

[B23] Fu L. Niu B. Zhu Z. Wu S. Li W. (2012). CD-HIT: Accelerated for clustering the next-generation sequencing data. *Bioinformatics* 28 3150–3152. 10.1093/bioinformatics/bts565 23060610 PMC3516142

[B24] Gou W. Fu Y. Yue L. Chen G. D. Cai X. Shuai M. et al. (2021). Gut microbiota, inflammation, and molecular signatures of host response to infection. *J. Genet. Genomics* 48 792–802. 10.1016/j.jgg.2021.04.002 34257044

[B25] Gurevich A. Saveliev V. Vyahhi N. Tesler G. (2013). QUAST: Quality assessment tool for genome assemblies. *Bioinformatics* 29 1072–1075. 10.1093/bioinformatics/btt086 23422339 PMC3624806

[B26] Harris C. R. Millman K. J. van der Walt S. J. Gommers R. Virtanen P. Cournapeau D. et al. (2020). Array programming with NumPy. *Nature* 585 357–362. 10.1038/s41586-020-2649-2 32939066 PMC7759461

[B27] Hill C. Guarner F. Reid G. Gibson G. R. Merenstein D. J. Pot B. et al. (2014). Expert consensus document. The international scientific association for probiotics and prebiotics consensus statement on the scope and appropriate use of the term probiotic. *Nat. Rev. Gastroenterol. Hepatol.* 11 506–514. 10.1038/nrgastro.2014.66 24912386

[B28] Hunter J. D. (2007). Matplotlib: A 2D graphics environment. *Comput. Sci. Eng.* 9 90–95. 10.1109/MCSE.2007.55

[B29] Jain C. Rodriguez-R L. M. Phillippy A. M. Konstantinidis K. T. Aluru S. (2018). High throughput ANI analysis of 90K prokaryotic genomes reveals clear species boundaries. *Nat. Commun.* 9:5114. 10.1038/s41467-018-07641-9 30504855 PMC6269478

[B30] Jaroszewski L. Li Z. Krishna S. S. Bakolitsa C. Wooley J. Deacon A. M. et al. (2009). Exploration of uncharted regions of the protein universe. *PLoS Biol.* 7:e1000205. 10.1371/journal.pbio.1000205 19787035 PMC2744874

[B31] Jones P. Binns D. Chang H. Y. Fraser M. Li W. McAnulla C. et al. (2014). InterProScan 5: Genome-scale protein function classification. *Bioinformatics* 30 1236–1240. 10.1093/bioinformatics/btu031 24451626 PMC3998142

[B32] Kanehisa M. Furumichi M. Tanabe M. Sato Y. Morishima K. (2017). KEGG: New perspectives on genomes, pathways, diseases and drugs. *Nucleic Acids Res.* 45 D353–D361. 10.1093/nar/gkw1092 27899662 PMC5210567

[B33] Kingma D. P. Welling M. (2014). *Auto-Encoding Variational Bayes.* Rio de Janeiro: ICLR.

[B34] Klotz C. Goh Y. J. O’Flaherty S. Barrangou R. (2020). S-layer associated proteins contribute to the adhesive and immunomodulatory properties of *Lactobacillus acidophilus* NCFM. *BMC Microbiol.* 20:248. 10.1186/s12866-020-01908-2 32787778 PMC7425073

[B35] Kokhlikyan N. Miglani V. Martin M. Wang E. Alsallakh B. Reynolds J. et al. (2020). Captum: A unified and generic model interpretability library for PyTorch. *arXiv* [Preprint] 10.48550/arXiv.2009.07896

[B36] Krogh A. Larsson B. von Heijne G. Sonnhammer E. L. (2001). Predicting transmembrane protein topology with a hidden Markov model: Application to complete genomes. *J. Mol. Biol.* 305 567–580. 10.1006/jmbi.2000.4315 11152613

[B37] Lieven C. Beber M. E. Olivier B. G. Bergmann F. T. Ataman M. Babaei P. et al. (2020). MEMOTE for standardized genome-scale metabolic model testing. *Nat. Biotechnol.* 38 272–276. 10.1038/s41587-020-0446-y 32123384 PMC7082222

[B38] Lin Z. Akin H. Rao R. Hie B. Zhu Z. Lu W. et al. (2023). Evolutionary-scale prediction of atomic-level protein structure with a language model. *Science* 379 1123–1130. 10.1126/science.ade2574 36927031

[B39] Louis P. Flint H. J. (2017). Formation of propionate and butyrate by the human colonic microbiota. *Environ. Microbiol.* 19 29–41. 10.1111/1462-2920.13589 27928878

[B40] Lundberg S. M. Lee S. I. (2017). “A unified approach to interpreting model predictions,” *Proceedings of the 31st International Conference on Neural Information Processing Systems* (Red Hook, NY: Curran Associates Inc.), 4768–4777.

[B41] Machado D. Andrejev S. Tramontano M. Patil K. R. (2018). Fast automated reconstruction of genome-scale metabolic models for microbial species and communities. *Nucleic Acids Res.* 46 7542–7553. 10.1093/nar/gky537 30192979 PMC6125623

[B42] Makhorin A. (2012). *GLPK (GNU Linear Programming Kit).* Boston, MA: Free Software Foundation.

[B43] McInnes L. Healy J. Saul N. Grossberger L. (2018). UMAP: Uniform manifold approximation and projection. *arXiv* [Preprint] 10.48550/arXiv.1802.03426

[B44] McKinney W. (2010). “Data structures for statistical computing in Python,” in *Proceedings of the 9th Python in Science Conference*, Vol. 445 Austin, 51–56. 10.25080/Majora-92bf1922-00a

[B45] Megchelenbrink W. Huynen M. Marchiori E. (2014). *optGpSampler*: An improved tool for uniformly sampling the solution-space of genome-scale metabolic networks. *PLoS One* 9:e86587. 10.1371/journal.pone.0086587 24551039 PMC3925089

[B46] Merkel D. (2014). Docker: Lightweight Linux containers for consistent development and deployment. *Linux J.* 2014:2.

[B47] Milani C. Duranti S. Bottacini F. Casey E. Turroni F. Mahony J. et al. (2017). The first microbial colonizers of the human gut: Composition, activities, and health implications of the infant gut microbiota. *Microbiol. Mol. Biol. Rev.* 81:e00036-17. 10.1128/MMBR.00036-17 29118049 PMC5706746

[B48] Mistry J. Chuguransky S. Williams L. Qureshi M. Salazar G. A. Sonnhammer E. L. L. et al. (2021). Pfam: The protein families database in 2021. *Nucleic Acids Res.* 49 D412–D419. 10.1093/nar/gkaa913 33125078 PMC7779014

[B49] Moher D. Liberati A. Tetzlaff J. Altman D. G. (2009). Preferred reporting items for systematic reviews and meta-analyses: The PRISMA statement. *PLoS Med.* 6:e1000097. 10.1371/journal.pmed.1000097 19621072 PMC2707599

[B50] Page A. J. Cummins C. A. Hunt M. Wong V. K. Reuter S. Holden M. T. et al. (2015). Roary: Rapid large-scale prokaryote pan genome analysis. *Bioinformatics* 31 3691–3693. 10.1093/bioinformatics/btv421 26198102 PMC4817141

[B51] Parks D. H. Imelfort M. Skennerton C. T. Hugenholtz P. Tyson G. W. (2015). CheckM: Assessing the quality of microbial genomes recovered from isolates, single cells, and metagenomes. *Genome Res.* 25 1043–1055. 10.1101/gr.186072.114 25977477 PMC4484387

[B52] Pasolli E. Asnicar F. Manara S. Zolfo M. Karcher N. Armanini F. et al. (2019). Extensive unexplored human microbiome diversity revealed by over 150,000 genomes from metagenomes spanning age, geography, and lifestyle. *Cell* 176 649–662.e20. 10.1016/j.cell.2019.01.001 30661755 PMC6349461

[B53] Paszke A. Gross S. Massa F. Lerer A. Bradbury J. Chanan G. et al. (2019). PyTorch: An imperative style, high-performance deep learning library. *ArXiv* [Preprint] 10.48550/arXiv.1912.01703

[B54] Pedregosa F. Varoquaux G. Gramfort A. Michel V. Thirion B. Grisel O. et al. (2011). Scikit-learn: Machine learning in Python. *J. Mach. Learn. Res.* 12 2825–2830.

[B55] Radicioni M. Koirala R. Fiore W. Leuratti C. Guglielmetti S. Arioli S. (2019). Survival of L. casei DG§(*Lactobacillus paracasei* CNCMI1572) in the gastrointestinal tract of a healthy paediatric population. *Eur. J. Nutr.* 58 3161–3170. 10.1007/s00394-018-1860-5 30498868 PMC6842349

[B56] Ranadheera C. S. Vidanarachchi J. K. Rocha R. S. Cruz A. G. Ajlouni S. (2017). Probiotic delivery through fermentation: Dairy vs. non-dairy beverages. *Fermentation* 3:67. 10.3390/fermentation3040067

[B57] Reiser P. Neubert M. Eberhard A. Torresi L. Zhou C. Shao C. et al. (2022). Graph neural networks for materials science and chemistry. *Commu. Mater.* 3:93. 10.1038/s43246-022-00315-6 36468086 PMC9702700

[B58] Rives A. Meier J. Sercu T. Goyal S. Lin Z. Liu J. et al. (2021). Biological structure and function emerge from scaling unsupervised learning to 250 million protein sequences. *Proc. Natl. Acad. Sci. U. S. A.* 118:e2016239118. 10.1073/pnas.2016239118 33876751 PMC8053943

[B59] Sanders M. E. Merenstein D. J. Reid G. Gibson G. R. Rastall R. A. (2019). Probiotics and prebiotics in intestinal health and disease: From biology to the clinic. *Nat. Rev. Gastroenterol. Hepatol.* 16 605–616. 10.1038/s41575-019-0173-3 31296969

[B60] Seemann T. (2014). Prokka: Rapid prokaryotic genome annotation. *Bioinformatics* 30 2068–2069. 10.1093/bioinformatics/btu153 24642063

[B61] Siezen R. J. van Hylckama Vlieg J. E. (2011). Genomic diversity and versatility of *Lactobacillus plantarum*, a natural metabolic engineer. *Microb. Cell Fact.* 10 (Suppl. 1):S3. 10.1186/1475-2859-10-S1-S3 21995294 PMC3271238

[B62] Suez J. Zmora N. Segal E. Elinav E. (2019). The pros, cons, and many unknowns of probiotics. *Nat. Med.* 25 716–729. 10.1038/s41591-019-0439-x 31061539

[B63] Tesson F. Hervé A. Mordret E. Touchon M. d’Humières C. Cury J. et al. (2022). Systematic and quantitative view of the antiviral arsenal of prokaryotes. *Nat. Commun.* 13:2561. 10.1038/s41467-022-30269-9 35538097 PMC9090908

[B64] Teufel F. Almagro Armenteros J. J. Johansen A. R. Gíslason M. H. Pihl S. I. Tsirigos K. D. et al. (2022). SignalP 6.0 predicts all five types of signal peptides using protein language models. *Nat. Biotechnol.* 40 1023–1025. 10.1038/s41587-021-01156-3 34980915 PMC9287161

[B65] The UniProt Consortium (2023). UniProt: The universal protein knowledgebase in 2023. *Nucleic Acids Res.* 51 D523–D531. 10.1093/nar/gkac1052 36408920 PMC9825514

[B66] Tierney B. T. Yang Z. Luber J. M. Beaudin M. Wibowo M. C. Baek C. et al. (2019). The landscape of genetic content in the gut and oral human microbiome. *Cell Host Microbe* 26 283–295.e8. 10.1016/j.chom.2019.07.008 31415755 PMC6716383

[B67] Topçuoğlu B. D. Lesniak N. A. Ruffin M. T. Wiens J. Schloss P. D. (2020). A framework for effective application of machine learning to microbiome-based classification problems. *mBio* 11:e00434-20. 10.1128/mBio.00434-20 32518182 PMC7373189

[B68] Turroni F. Milani C. Duranti S. Mahony J. van Sinderen D. Ventura M. (2018). Glycan utilization and cross-feeding activities by bifidobacteria. *Trends Microbiol.* 26 339–350. 10.1016/j.tim.2017.10.001 29089173

[B69] Van Rossum G. Drake F. L. (2009). *Python 3 Reference Manual.* Scotts Valley, CA: CreateSpace.

[B70] Wilkerson M. D. Hayes D. N. (2010). ConsensusClusterPlus: A class discovery tool with confidence assessments and item tracking. *Bioinformatics* 26 1572–1573. 10.1093/bioinformatics/btq170 20427518 PMC2881355

[B71] Wolf T. Debut L. Sanh V. Chaumond J. Delangue C. Moi A. et al. (2020). “Transformers: State-of-the-art natural language processing,” in *Proceedings of the 2020 Conference on Empirical Methods in Natural Language Processing: System Demonstrations*, (Stroudsburg, PA: Association for Computational Linguistics), 38–45.

[B72] Wootton J. C. Federhen S. (1996). Analysis of compositionally biased regions in sequence databases. *Methods Enzymol.* 266 554–571. 10.1016/s0076-6879(96)66035-2 8743706

[B73] Zhang Y. Zhou M. Zhou Y. Guan X. (2023). Dietary components regulate chronic diseases through gut microbiota: A review. *J. Sci. Food Agric.* 103 6752–6766. 10.1002/jsfa.12732 37225671

[B74] Zhang Z. Zhang Q. Wang T. Xu N. Lu T. Hong W. et al. (2022). Assessment of global health risk of antibiotic resistance genes. *Nat. Commun.* 13:1553. 10.1038/s41467-022-29283-8 35322038 PMC8943045

[B75] Zitnik M. Nguyen F. Wang B. Leskovec J. Goldenberg A. Hoffman M. M. (2019). Machine learning for integrating data in biology and medicine: Principles, practice, and opportunities. *Inf. Fusion* 50 71–91. 10.1016/j.inffus.2018.09.012 30467459 PMC6242341

[B76] Zmora N. Suez J. Elinav E. (2019). You are what you eat: Diet, health and the gut microbiota. *Nat. Rev. Gastroenterol. Hepatol.* 16 35–56. 10.1038/s41575-018-0061-2 30262901

[B77] Zmora N. Zilberman-Schapira G. Suez J. Mor U. Dori-Bachash M. Bashiardes S. et al. (2018). Personalized gut mucosal colonization resistance to empiric probiotics is associated with unique host and microbiome features. *Cell* 174 1388–1405.e21. 10.1016/j.cell.2018.08.041 30193112

