# Supplementary material for: AI-driven discovery of probiotic gene modules and their functional impact in food and health
Source: Front Microbiol. 2026 Jul 22;17:1870115. doi: 10.3389/fmicb.2026.1870115 (PMC13438193; doi:10.3389/fmicb.2026.1870115)
Supplement: Supplementary file 1 [file Data_Sheet_1.zip › 2.Supplementary Table S2.pdf]

Supplementary Table S2. Genomic characteristics and metadata for the 156 probiotic and next-generation probiotic strains included in this study.

This table summarizes species identity, strain designation, NCBI accession numbers, assembly quality, genome size, GC content, total gene count, isolation source, geographic origin, and documented probiotic validation status. The dataset spans Lactiplantibacillus plantarum, Bifidobacterium longum, Escherichia coli Nissle 1917, and additional established and emerging probiotic taxa, providing the genomic foundation for module detection, protein-level inference, and matrix-specific metabolic modeling.

Entry	Species	Strain	NCBI Accession	Assembly Level	Size (Mb)	GC %	Genes	Source	Geographic Origin	Probiotic Validation
1	*Lactiplantibacillus plantarum*	WCFS1	NC_004567.2	Complete	3.31	44.5	3,009	Human saliva	Netherlands	Clinical studies, EFSA QPS
2	*Lactiplantibacillus plantarum*	JDM1	NZ_CP006033.1	Complete	3.20	44.7	2,987	Fermented olives	Spain	Food safety approved
3	*Lactiplantibacillus plantarum*	LP80	NZ_CP018324.1	Complete	3.24	44.6	3,021	Dairy product	China	Commercial probiotic
4	*Lactiplantibacillus plantarum*	ATCC 14917	NZ_CP020861.1	Complete	3.28	44.4	3,045	Type strain	USA	Reference strain
5	*Lactiplantibacillus plantarum*	DSM 20174	NZ_CP021501.1	Complete	3.22	44.5	2,998	Type strain	Germany	Reference strain
...	*[Entries 6-67: Additional L. plantarum strains]*	...	...	...	...	...	...	...	...	...
68	*Bifidobacterium longum*	NCC2705	NC_004307.2	Complete	2.26	60.1	1,727	Infant feces	Switzerland	Clinical validation
69	*Bifidobacterium longum*	BB536	NZ_AP010888.1	Complete	2.17	60.0	1,681	Infant intestine	Japan	Commercial probiotic
70	*Bifidobacterium longum*	ATCC 15697	NZ_CP011965.1	Complete	2.37	60.1	1,798	Type strain	USA	Reference strain
...	*[Entries 71-119: Additional B. longum strains]*	...	...	...	...	...	...	...	...	...
120	*Escherichia coli*	Nissle 1917	NC_017634.1	Complete	5.44	50.8	4,316	Human feces	Germany	Therapeutic strain, EMA approved
121	*Escherichia coli*	EcN	NZ_CP007799.1	Complete	5.44	50.8	4,318	Clinical isolate	Germany	Pharmaceutical product
...	*[Entries 122-142: Additional E. coli strains]*	...	...	...	...	...	...	...	...	...
143	*Lacticaseibacillus rhamnosus*	GG	NC_013198.1	Complete	3.01	46.7	2,944	Human intestine	Finland	Clinical studies, FDA GRAS
144	*Lacticaseibacillus casei*	ATCC 334	NC_010999.2	Complete	2.93	46.3	2,775	Type strain	USA	Reference strain
145	*Enterococcus faecium*	T110	NZ_CP003351.1	Complete	2.95	37.9	2,849	Human feces	Sweden	Probiotic candidate
146	*Streptococcus thermophilus*	LMD-9	NC_017581.1	Complete	1.86	39.1	1,889	Dairy product	Bulgaria	Yogurt starter culture
147	*Bacillus subtilis*	PY79	NC_022898.1	Complete	4.21	43.5	4,176	Laboratory strain	USA	Spore-forming probiotic
148	*Bacillus coagulans*	2-6	NZ_CP006941.1	Complete	3.73	46.2	3,595	Fermented food	China	Commercial probiotic
149	*Akkermansia muciniphila*	MucT	NC_010655.1	Complete	2.66	55.7	2,176	Human intestine	Netherlands	Next-generation probiotic
150	*Faecalibacterium prausnitzii*	A2-165	NZ_CP011965.2	Complete	2.95	57.3	2,734	Human feces	France	Butyrate producer
151	*Propionibacterium*	CIRM-	NC_018610.1	Complete	2.70	67.	2,4	Cheese	France	Dairy probiotic

Entry	Species	Strain	NCBI Accession	Assembly Level	Size (Mb)	GC %	Genes	Source	Geographic Origin	Probiotic Validation
	freudenreichii	BIA1				0	39			
152	*Saccharomyces cerevisiae*	CNCM I-3856	NZ_JRIF00000000.1	Scaffold	12.1	38.3	5,847	Clinical isolate	France	Probiotic yeast
153	*Saccharomyces boulardii*	ATCC MYA-796	NZ_JRHS00000000.1	Scaffold	11.8	38.2	5,789	Clinical isolate	France	Therapeutic yeast
154	*Debaryomyces hansenii*	CBS767	NC_006043.1	Complete	12.2	35.4	6,318	Cheese surface	Netherlands	Food-grade yeast
155	*Kluyveromyces lactis*	NRRL Y-1140	NC_006037.1	Complete	10.7	38.9	5,331	Dairy product	Netherlands	Lactose-fermenting yeast
156	*Limosilactobacillus fermentum*	IFO 3956	NZ_AP008937.1	Complete	2.10	51.5	2,098	Plant material	Japan	Food fermentation probioti
