# Supplementary material for: AI-driven discovery of probiotic gene modules and their functional impact in food and health
Source: Front Microbiol. 2026 Jul 22;17:1870115. doi: 10.3389/fmicb.2026.1870115 (PMC13438193; doi:10.3389/fmicb.2026.1870115)
Supplement: Supplementary file 1 [file Data_Sheet_1.zip › 3.Supplementary Table S3_.pdf]

Supplementary Table S3. Annotated gene modules identified across 156 probiotic genomes

Core modules were defined as present in ≥95% of genomes. Matrix enrichment indicates statistically significant overrepresentation under matrix-constrained metabolic simulations.

Module_ID	Gene_or_Locus_Tag	Functional_Domain	Functional_Description	Prevalence (%)	Core_or_Accessory	Associated_Pathways	Matrix_Enrichment	Notes
GM001	mucBP	Adhesion and Colonization	Mucin-binding protein	98%	Core	KO: K13923	Dairy	Highly conserved
GM002	fbpA	Adhesion and Colonization	Fibronectin-binding protein	96%	Core	UniProt: Q9ZC12	Not enriched	—
GM003	spaCBA	Adhesion and Colonization	Pilus assembly operon	72%	Accessory	KO: K13510	Dairy	Strain-variable
GM004	groESL	Stress Resistance	Chaperone complex	100%	Core	KO: K04077	Dairy	Strong activation in dairy
GM005	gadB–gadC	Stress Resistance	Glutamate decarboxylase system	97%	Core	KO: K01580	Dairy	Acid tolerance
GM006	bsh	Stress Resistance	Bile salt hydrolase	88%	Accessory	KO: K01442	Dairy	PLM-validated
GM007	ackA	SCFA Biosynthesis	Acetate kinase	81%	Accessory	KO: K00925	Plant	SCFA flux driver
GM008	ptb	SCFA Biosynthesis	Phosphate butyryltransferase	79%	Accessory	KO: K00634	Plant	Butyrate pathway
GM009	plnA	Antimicrobial Activity	Plantaricin precursor	41%	Accessory	UniProt: Q88ZP3	Fermented	Bacteriocin cluster
GM010	nisin operon	Antimicrobial Activity	Nisin biosynthesis operon	22%	Accessory	KO: K20492	Fermented	Enriched in competitive strains
