# Supplementary material for: AI-driven discovery of probiotic gene modules and their functional impact in food and health
Source: Front Microbiol. 2026 Jul 22;17:1870115. doi: 10.3389/fmicb.2026.1870115 (PMC13438193; doi:10.3389/fmicb.2026.1870115)
Supplement: Supplementary file 1 [file Data_Sheet_1.zip › 4. Supplementary Table S4_.pdf]

Supplementary Table S4. Machine-Learning Hyperparameters and Evaluation Procedures

Model	Hyperparameters	Optimization Method	Training / Validation Split	Random Seed	Feature-Selection Method
**XGBoost (IL-10 induction, survivability)**	500 estimators; learning rate = 0.05; max depth = 6; subsample = 0.8; colsample_bytree = 0.7; min_child_weight = 3; γ = 0.1; objective = “binary:logistic”; evaluation metric = AUC	Optuna (100 trials)	80% training / 20% held-out test; stratified 5-fold CV	42	ANOVA F-test ($p < 0.05$); Recursive Feature Elimination (100 iterations); L1-regularized logistic regression
**Random Forest (SCFA flux prediction)**	1,000 trees; unlimited depth; min samples split = 2; min samples leaf = 1; feature sampling = sqrt	Default scikit-learn optimization	80% training / 20% test; 5-fold CV	42	Same three-stage feature-selection pipeline
**Neural Network (auxiliary classification)**	3 dense layers (256–128–64 units); ReLU activation; dropout = 0.3; batch size = 32; Adam optimizer (LR = 1×10^{-3}); early stopping (patience = 20 epochs)	Manual tuning + early stopping	80% training / 20% test; 5-fold CV	42	Same three-stage feature-selection pipeline
**Shared Preprocessing (all models)**	PCA retaining ≥95% variance; KNN imputation (k = 5); isolation forest (contamination = 0.02); z-score normalization	—	Applied before all model training	42	Filtering of gene modules (<5% prevalence removed); metabolic flux threshold $<1\times10^{-6}$ removed
