# Supplementary material for: AI-driven discovery of probiotic gene modules and their functional impact in food and health
Source: Front Microbiol. 2026 Jul 22;17:1870115. doi: 10.3389/fmicb.2026.1870115 (PMC13438193; doi:10.3389/fmicb.2026.1870115)
Supplement: Supplementary file 1 [file Data_Sheet_1.zip › 5. Supplementary Table S5.pdf]

Supplementary Table S5. Performance comparison of PLM predictions with traditional annotation tools

Method	Precision	Recall	F1-Score	AUC-ROC	Matthews CC
**PLM (ESM-2 + ProtT5)**	**0.89**	**0.85**	**0.87**	**0.92**	**0.74**
InterProScan	0.82	0.71	0.76	0.84	0.63
HMMER	0.78	0.69	0.73	0.81	0.58
BLAST+	0.75	0.82	0.78	0.83	0.61
Pfam	0.81	0.64	0.71	0.79	0.56
eggNOG-mapper	0.73	0.76	0.74	0.80	0.55
DeepFRI	0.84	0.79	0.81	0.87	0.68
